# The neurological level of spinal cord injury and cardiovascular risk factors: a systematic review and meta-analysis

**DOI:** 10.1038/s41393-021-00678-6

**Published:** 2021-08-20

**Authors:** Peter Francis Raguindin, Gion Fränkl, Oche Adam Itodo, Alessandro Bertolo, Ramona Maria Zeh, Simona Capossela, Beatrice Minder, Jivko Stoyanov, Gerold Stucki, Oscar H. Franco, Taulant Muka, Marija Glisic

**Affiliations:** 1 Swiss Paraplegic Research, Nottwil, Switzerland; 2 Institute of Social and Preventive Medicine (ISPM), University of Bern, Bern, Switzerland; 3 Graduate School for Health Sciences, University of Bern, Bern, Switzerland; 4 Graduate School for Cellular and Biomedical Sciences, University of Bern, Bern, Switzerland; 5 Public Health & Primary Care Library, University Library of Bern,, University of Bern, Bern, Switzerland

**Keywords:** Risk factors, Epidemiology

## Abstract

**Study design:**

Systematic review and meta-analysis.

**Objective:**

To determine the difference in cardiovascular risk factors (blood pressure, lipid profile, and markers of glucose metabolism and inflammation) according to the neurological level of spinal cord injury (SCI).

**Methods:**

We searched 5 electronic databases from inception until July 4, 2020. Data were extracted by two independent reviewers using a pre-defined data collection form. The pooled effect estimate was computed using random-effects models, and heterogeneity was calculated using I^2^ statistic and chi-squared test (CRD42020166162).

**Results:**

We screened 4863 abstracts, of which 47 studies with 3878 participants (3280 males, 526 females, 72 sex unknown) were included in the meta-analysis. Compared to paraplegia, individuals with tetraplegia had lower systolic and diastolic blood pressure (unadjusted weighted mean difference, −14.5 mmHg, 95% CI −19.2, −9.9; −7.0 mmHg 95% CI −9.2, −4.8, respectively), lower triglycerides (−10.9 mg/dL, 95% CI −19.7, −2.1), total cholesterol (−9.9 mg/dL, 95% CI −14.5, −5.4), high-density lipoprotein (−1.7 mg/dL, 95% CI −3.3, −0.2) and low-density lipoprotein (−5.8 mg/dL, 95% CI −9.0, −2.5). Comparing individuals with high- vs. low-thoracic SCI, persons with higher injury had lower systolic and diastolic blood pressure (−10.3 mmHg, 95% CI −13.4, −7.1; −5.3 mmHg 95% CI −7.5, −3.2, respectively), while no differences were found for low-density lipoprotein, serum glucose, insulin, and inflammation markers. High heterogeneity was partially explained by age, prevalent cardiovascular diseases and medication use, body mass index, sample size, and quality of studies.

**Conclusion:**

In SCI individuals, the level of injury may be an additional non-modifiable cardiovascular risk factor. Future well-designed longitudinal studies with sufficient follow-up and providing sex-stratified analyses should confirm our findings and explore the role of SCI level in cardiovascular health and overall prognosis and survival.

## Introduction

Individuals with spinal cord injury (SCI) are known to have a higher metabolic and cardiovascular disease (CVD) risk in comparison to able-bodied individuals (ABI) due to changes in metabolism, body morphology, and relative inactivity following the injury [1–3]. Long-term studies have shown that cardiovascular-related mortality is higher compared to the general population and that CVDs are one of the leading causes of death in individuals with traumatic and non-traumatic SCI [4]. Using the data from the general population, cohort studies have identified several non-modifiable (e.g., sex, age, genetic predisposition, etc.) and modifiable (e.g., blood pressure, serum lipid profile, glucose levels, etc.) determinants of increased cardiovascular risk [5]. CVD risk prediction scoring systems such as Framingham risk score (FRS) or the HEART score have been developed combining known CVD risk determinants, and these scores are widely used for risk stratification in the general population [6, 7]. However, the determinants of increased CVD risk in SCI have not been fully elucidated, and the use of such prediction models in SCI have been rarely studied in the literature [8–10]. FRS may underestimate the CVD disease risk in people with SCI [8–10] as this scoring system does not include SCI-specific risk factors. The level of injury may be an additional non-modifiable factor that may aggravate CVD risk and, therefore, a potential factor to consider in the development of SCI-specific CVD risk screening tools.

Biologically, the level of injury could significantly affect the disability and  functioning of an individual, which determines mobility and baseline activity [11]. Depending on the injury level, autonomic control, and metabolism of hormones, glucose, lipid, and catecholamine could be impaired additionally [2, 3, 12]. Despite numerous attempts to determine the association between the anatomic level of the injury and CVD risk factors, the majority of studies were insufficiently powered to capture the changes in CVD risk factors according to injury level [13–15]. Previous narrative reviews had limited methodological quality and previous systematic reviews focused on blood lipids and blood pressure only [12, 16, 17]. Thus, the evidence on the influence of injury level and CVD risk factors is still limited.

In this review, we aim to summarize the available evidence on the differences of modifiable cardiovascular risk factors according to SCI level and explore further how completeness and injury duration may affect this association. In addition, we aim to identify the current literature gaps and provide directions for future research.

## Methods

### Data sources and search strategy

The current review was conducted following a recently published guideline on how to perform systematic reviews and meta-analysis in medical research and reported according to the PRISMA (Preferred Reporting Items for Systematic Reviews and Meta-Analyses) checklist [18]. The protocol was registered in the PROSPERO Database (Registration number CRD42020166162).

An experienced information specialist searched four bibliographic databases: MEDLINE (National Library of Medicine, US), EMBASE (Elsevier, Netherlands), Web of Science (Clarivate Analytics, US), and Cochrane (Cochrane Collaboration, UK) from inception until July 4, 2020 (date last searched). In addition, we downloaded the first 400 results from Google Scholar. The search strategy combined terms related to (i) spinal cord injury such as “spinal cord injury,” “paraplegia,” “quadriplegia,” etc., and (ii) CVD risk factors (e.g., lipoproteins, inflammation markers, etc.) and was filtered to include human studies only. Further, the reference lists of the included studies and relevant reviews were searched for eligible studies. Details of the search strategy are provided in the Appendix.

### Study selection and eligibility criteria

We limited our search to observational studies conducted in adult (≥18 years old) humans with SCI. In particular, observational studies were eligible for inclusion if they provided information on any of the following CVD risk factors; blood pressure, glucose metabolism, serum lipids, inflammation markers, coagulation system/fibrinolysis markers, homocysteine, markers of oxidative stress, markers of endothelial dysfunction and vascular function and carotid atherosclerosis, disaggregated by different levels of SCI. We excluded studies without comparison groups (e.g., studies focusing solely on individuals with paraplegia), animal and in-vitro studies, reviews, commentaries, case studies, letters to the editor, and conference abstracts. We used no language restrictions.

Two authors screened titles and abstracts using the inclusion and exclusion criteria mentioned above. Afterwards, the full texts of the identified articles were reviewed independently by two authors. Disagreements on the decision for inclusion were discussed between the authors, and if consensus was not reached, a third author was called to adjudicate the decision. Reasons for exclusion of articles that underwent full-text reviews were tabulated.

### Data extraction

We used a predesigned data collection form to collect relevant information from identified articles. To ensure the accuracy of data extraction, two authors independently extracted the data from identified articles. For variables/outcomes expressed in SI (Système Internationale), we converted the data, using the standard conversion tables (US National Institute of Standards and Measures), to conventional units. In studies where median and ranges (interquartile range, minimum-maximum values, crude range) were reported, we converted the figures into mean and standard deviation. Outcomes were collected according to different injury levels. For cases of multiple publications, the most recent information or the publication with the most relevant outcome was used for the data extraction.

### Risk of bias assessment

Methodological quality was assessed independently by two independent authors using the Newcastle-Ottawa Scale (NOS) for case-control, cross-sectional, or cohort studies, as applicable to the study [19]. The scale was developed for the assessment of non-randomized and observational studies, and assesses each study based on three broad categories: selection of the study groups or participants, the comparability of the groups, and the ascertainment of either the exposure or outcome of interest. Quality of studies was assessed and graded highest based on specific parameters mentioned, and higher-quality studies were accorded with higher points (8–10, good quality; 5–7 fair quality; <5 low quality).

### Data synthesis and analysis

Outcomes were grouped into four (4) major categories, namely: (a) blood pressure (systolic blood pressure, diastolic blood pressure, mean arterial pressure), (b) lipids (triglycerides, total cholesterol, high-density lipoprotein [HDL], low-density lipoprotein [LDL], HDL/LDL ratio, total cholesterol/HDL ratio), (c) markers of glucose metabolism (serum glucose, insulin, Homeostatic Model Assessment for Insulin Resistance [HOMA-IR]), and (d) inflammation markers (Creactive protein (CRP and high-sensitivity CRP), tumor necrosis factor-alpha and interleukin 6). All outcome measures were expressed in conventional units. We computed pooled means and standard deviation, and weighted mean difference based on the extracted measurement from each study. We combined the mean difference by weight using the random-effects model developed by Der Simonian and Laird. In the random-effects model, weighted means and mean difference accounts for both the within-study and between-studies errors as opposed to the usual computation of merely using errors (sample size). Thus, we allow that the true effect estimate is within a distribution rather than having one true effect estimate. Weighted means were deemed appropriate as all our outcomes were expressed in similar scales (Standardized mean difference would be appropriate if different scales were used, which did not hold true in our review). Heterogeneity was assessed using the Cochrane χ² statistic and the I² statistic. Study characteristics including patient characteristics (median age, median body mass index, sex distribution, and baseline cardiovascular disease/medications), injury characteristics (completeness of lesion and duration of injury) and study characteristics (study location, the median number of participants, and study quality) were pre-specified as characteristics for assessment of heterogeneity and were evaluated using stratified analyses and random-effects meta-regression if eight or more studies were included in the meta-analysis. In addition, we performed meta-regression to determine the association between the age, percentage of the male population, injury duration, completeness of injury, and body mass index to each cardiovascular risk factor. To assess the impact of each study in the analyses, a leave-one-out analysis was done by iteratively estimating the weighted mean difference by removing a study one at a time. Asymmetry was assessed by Egger’s test, and publication bias was evaluated through a funnel plot. All tests were performed by using two-tailed tests, with *p* < 0.05 as significant. All statistical analyses were conducted with STATA, Release 16.1 (Stata Corp, College Station, Texas, USA). The studies that could not be quantitatively pooled were descriptively summarized.

## Results

### Search results

**Fig. 1:**
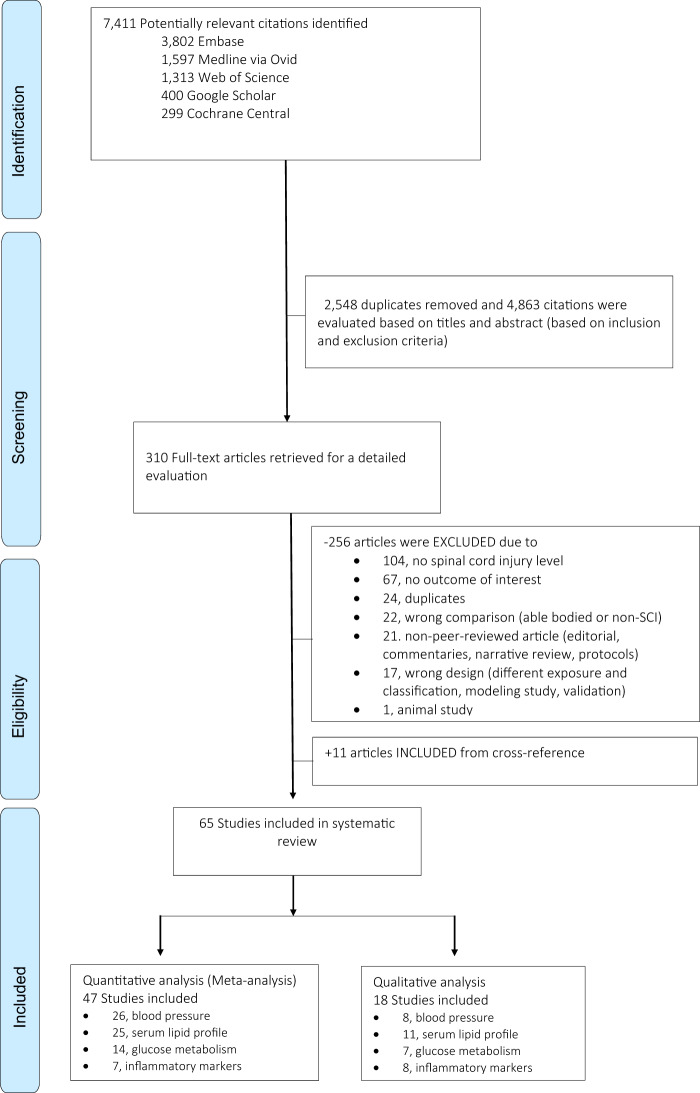
Flowchart of included studies in the systematic review.

A total of 7411 relevant citations were identified on 5 databases searched (Fig. 1). After removing duplicates, 4863 citations were evaluated using titles and abstracts by two independent reviewers for relevance. Three hundred ten (310) articles underwent full-text review, and 11 additional articles were identified from cross-reference. In total, 65 observational studies comprising 7342 individuals with SCI were included in the review, of which 47 studies contributed to the meta-analysis with 3878 participants (3280 males, 526 females, 72 unknown sex). We were able to meta-analyze unadjusted mean biomarker levels from 26 studies on blood pressure, 25 on serum lipid profiles, 14 on markers of glucose metabolism, and 7 on inflammation markers. The majority of studies (*n* = 44, 93.6%) compared individuals with tetraplegia and paraplegia, while 12 studies compared high- to low-paraplegia (T6 as the level of discrimination). All studies were performed in individuals with chronic SCI (median SCI duration was 13.5 years, interquartile range 10.7–14.7 years) and among relatively young study participants (median age was 39.3 years), and 22 studies (46%) were conducted only in males. Thirty-eight studies (38, 80%) were of small sample size (*n* < 100). Most of the studies were done in the US and Canada (*n* = 26, 55%) and Europe (*n* = 10, 21%), while eight studies were done in Asia and one in Latin America (Brazil). Table 1 summarizes the characteristics of the studies included in the meta-analysis, and detailed information on study characteristics of all studies included in our review can be found in the Appendix (Appendix Table A1). The results and reasons for non-extraction of the other 18 studies (11 for serum lipids, 8 for inflammation markers, 7 markers for glucose metabolism, and 8 for blood pressure) were qualitatively described and summarized in the Appendix (Appendix Table A2). We only report in the text the weighted mean difference (WMD) for statistically significant values, otherwise, crude group mean was provided.

**Table 1:** Descriptive characteristics of studies included in the meta-analysis (*n* = 47).

Characteristic	No. of studies	References
Level of injury		
Tetraplegia vs paraplegia	44	Aadriansen 2016, Akbal 2013, Apstein 1998, Baumann 1992, Baumann 1999, Brenes 1986, Buchholz 2009, Campbell 2004, Cardus 1992, Davies 2007, de Groot 2008, Farkas 2018, Frost 2005, Gibson 2008, Gorgey 2010, Gorgey 2011, Grimm 1997, Heidebreder 1982, Houtmann 1999, Huang 2008, Janssen 1997, Katzelnick 2019, Kemp 2000, Kim 2016, King 1994, Kjaer 2001, Kooner 1988, Laclaustra 2014, Legramante 2001, Matos Souza 2010, Miyatani 2014, O Brien 2017, Raymond 2010, Sabour 2013, Schmid 2000, Schmid 2008, Wang 2007, Wecht 2001, Wecht 2006, Wong 2001, Yahiro 2019, Zhong 1995, Zhou 1997, Zhu 2013
High vs. low paraplegia	12	Aadriansen 2016, Bernardi 2019, Campbell 2004, Janssen 1997, Katzelnick 2017, Katzelnick 2019, Kim 2016, King 1994, Krum 1989, Raymond 2010, Sisto 2012, Zhu 2013
Proportion of complete SCI		
≤63% (median)	15	Akbal 2013, Davies 2007, de Groot 2008, Gibson 2008, Grimm 1997, Huang 2008, Janssen 1997, Katzelnick 2017, Katzelnick 2019, Laclaustra 2014, Miyatani 2014, Raymond 2010, Sabour 2013, Wecht 2001, Wecht 2006
>63%	15	Aadriansen 2016, Baumann 1999, Farkas 2018, Gorgey 2010, Gorgey 2011, Kemp 2000, Kooner 1988, Legramante 2001, O Brien 2017, Schmid 2000, Schmid 2008, Wang 2007, Wong 2001, Yahiro 2019, Zhou 1997
Duration of injury (years)		
≤13.5 y (median)	20	Akbal 2013, Baumann 1999, Cardus 1992, Davies 2007, Frost 2005, Grimm 1997, Huang 2008, Katzelnick 2017, Krum 1989, Matos Souza 2010 Miyatani 2014, O Brien 2017, Raymond 2010, Sabour 2013, Schmid 2000, Schmid 2008, Sisto 2012, Wang 2007, Wecht 2001, Wecht 2006
>13.5 y	15	Aadriansen 2016, Baumann 1992, Buchholz 2009, Cardus 1992, de Groot 2008, Farkas 2018, Gibson 2008, Janssen 1997, Katzelnick 2019, Kemp 2000, Laclaustra 2014, Wong 2001, Yahiro 2019, Zhong 1995, Zhu 2013
Sex		
Male only	22	Apstein 1998, Baumann 1992, Baumann 1999, Bernardi 2019, Campbell 2004, Cardus 1992, Frost 2005, Gorgey 2010, Gorgey 2011, Grimm 1997 Houtmann 1999, Janssen 1997, Kooner 1988, Legramante 2001, Matos Souza 2010, Matos Suoza 2010, O Brien 2017, Schmid 2000, Wang 2007, Wecht 2001, Wecht 2006, Yahiro 2019, Zhong 1995
Both sex	25	Aadriansen 2016, Brenes 1986, Buchholz 2009, Davies 2007, de Groot 2008, Farkas 2018, Gibson 2008, Heidebreder 1982, Huang 2008, Katzelnick 2017, Katzelnick 2019, Kemp 2000, Kim 2016, King 1994, Kjaer 2001, Krum 1989, Laclaustra 2014, Miyatani 2014, Raymond 2010, Sabour 2013, Schmid 2008, Sisto 2012, Wong 2001, Zhou 1997, Zhu 2013
Study size		
<50	21	Bernardi 2019, Campbell 2004, Farkas 2018, Frost 2005, Grimm 1997, Heidebreder 1982, Huang 2008, Janssen 1997, Katzelnick 2017, Kim 2016, King 1994, Kjaer 2001, Kooner 1988, Krum 1989, Legramante 2001, Matos Souza 2010, O Brien 2017, Raymond 2010, Wecht 2001, Wecht 2006, Zhou 1997
50–100	16	Akbal 2013, Apstein 1998, Baumann 1992, Baumann 1999, Brenes 1986, Buchholz 2009, Cardus 1992, Davies 2007, Gibson 2008, Gorgey 2011A, Gorgey 2011B, Miyatani 2014, Schmid 2000, Schmid 2008, Wong 2001, Wang 2007
>100	10	Aadriansen 2016, de Groot 2008, Katzelnick 2019, Kemp 2000, Laclaustra 2014, Sabour 2013, Sisto 2012, Yahiro 2019, Zhong 1995, Zhu 2013
Age, years		
≤39.3 y (median)	25	Akbal 2013, Baumann 1999, Bernardi 2019, Brenes 1986, Campbell 2004, Cardus 1992, Gorgey 2010, Gorgey 2011, Grimm 1997, Huang 2008, Janssen 1997, Katzelnick 2017, Kim 2016, King 1994, Krum 1989, Legramante 2001, Matos Souza 2010, O Brien 2017, Raymond 2010, Sabour 2013, Schmid 2000, Schmid 2008, Wang 2007, Wecht 2006, Zhou 1997
>39.3 y	20	Aadriansen 2016, Apstein 1998, Baumann 1992, Buchholz 2009, Cardus 1992, Davies 2007, de Groot 2008, Farkas 2018, Frost 2005, Gibson 2008, Katzelnick 2019, Kemp 2000, Laclaustra 2014, Miyatani 2014, Sisto 2012, Wecht 2001, Wong 2001, Yahiro 2019, Zhong 1995, Zhu 2013
BMI, kg/m^2^		
≤24.9 (median)	17	Aadriansen 2016, Baumann 1999, Brenes 1986, de Groot 2008, Gorgey 2010, Gorgey 2011, Janssen 1997, Kim 2016, Matos Souza 2010, O Brien 2017, Raymond 2010, Sabour 2013, Schmid 2000, Schmid 2008, Wang 2007, Wecht 2006, Wong 2001
>24.9	14	Akbal 2013, Baumann 1992, Buchholz 2009, Farkas 2018, Gibson 2008, Grimm 1997, Katzelnick 2017, Katzelnick 2019, Kemp 2000, Miyatani 2014, Wecht 2001, Yahiro 2019, Zhong 1995, Zhu 2013
Location		
Europe	11	Aadriansen 2016, Bernardi 2019, de Groot 2008, Heidebreder 1982, Janssen 1997, Kjaer 2001, Kooner 1988, Legramante 2001, Schmid 2000, Schmid 2008, Laclaustra 2014
North America	26	Apstein 1998, Baumann 1992, Baumann 1999, Brenes 1986, Buchholz 2009, Cardus 1992, Davies 2007, Farkas 2018, Frost 2005, Gibson 2008, Gorgey 2010, Gorgey 2011, Grimm 1997, Katzelnick 2017, Katzelnick 2019, Kemp 2000, Kim 2016, King 1994, Miyatani 2014, O Brien 2017, Sisto 2012, Wecht 2001, Wecht 2006, Yahiro 2019, Zhong 1995, Zhu 2013
South America	1	Matos Souza 2010
Asia	9	Akbal 2013, Campbell 2004, Huang 2008, Krum 1989, Raymond 2010, Sabour 2013, Wang 2007, Wong 2001, Zhou 1997
Outcomes		
Blood lipids	25	Akbal 2013, Apstein 1998, Baumann 1992, Bernardi 2019, Brenes 1986, Buchholz 2009, Cardus 1992, de Groot 2008, Farkas 2018, Gibson 2008, Gorgey 2010, Gorgey 2011, Janssen 1997, Kemp 2000, Lacluastra 2014, Matos Souza 2010, Miyatani 2014, O Brien 2017, Sabour 2013, Schmid 2000, Schmid 2008, Wang 2007, Wong, 2001, Yahiro 2019, Zhong 1995
Blood glucose	14	Akbal 2013, Baumann 1999, Buchholz 2009, Campbell 2004, Farkas 2018, Gibson 2008, Gorgey 2010, Huang 2008, Matos Souza 2010, Miyatani 2014, O Brien 2017, Sabour 2013, Yahiro 2019, Wang 2007
Blood pressure	26	Aadriansen 2016, Akbal 2013, Buchholz 2009, Farkas 2018, Grimm 1997, Heidebreder 1982, Houtmann 1999, Janssen 1997, Katzelnick 2017, Katzelnick 2019, Kim 2016, King 1994, Kjaer 2001, Kooner 1988, Krum 1989, Legramante 2001, Matos Souza 2010, Miyatani 2014, Raymond 2010, Sabour 2013, Sisto 2012, Wecht 2001, Wecht 2006, Yahiro 2019, Zhou 1997, Zhu 2013
Inflammation markers	7	Buchholz 2009, Davies 2007, Farkas 2018, Frost 2005, Gibson 2008, Huang 2008, Wang 2007
Study quality		
Moderate (5–7)	6	Baumann 1992, Campbell 2004, Cardus 1992, Heidebreder 1982, Kooner 1988, Zhou 1997
Good (8–10)	41	Aadriansen 2016, Akbal 2013, Apstein 1998, Baumann 1999, Bernardi 2019, Brenes 1986, Buchholz 2009, Davies 2007, de Groot 2008, Farkas 2018, Frost 2005, Gibson 2008, Gorgey 2010, Gorgey 2011, Grimm 1997, Huang 2008, Janssen 1997, Katzelnick 2017, Katzelnick 2019, Kemp 2000, Kim 2016, King 1994, Kjaer 2001, Krum 1989, Laclaustra 2014, Legramante 2001, Matos Souza 2010, Miyatani 2014, O Brien 2017, Raymond 2010, Sabour 2013, Schmid 2000, Schmid 2008, Sisto 2012, Wang 2007, Wecht 2001, Wecht 2006, Wong 2001, Yahiro 2019, Zhong 1995, Zhu 2013

### Blood pressure

Based on the findings from 20 studies and 1516 individuals with SCI, systolic and diastolic BP, and mean arterial pressures were lower in tetraplegia in comparison to individuals with paraplegia with the weighted mean difference (WMD) −14.5 mmHg (95% CI −19.2, −9.9, *p* < 0.001, I^2^ 92.5%), −7.0 mmHg (95% CI −9.2, −4.8, *p* < 0.001, I^2^ 79.7%) and −15.2 mmHg (95% CI −25.0, −5.4, *p* < 0.001, I^2^ 87.3%), respectively (Table 2). In line with this, based on findings from 10 studies and 761 SCI individuals, individuals with high-paraplegia had lower systolic and diastolic BP compared to low-paraplegia (at T5-T6 and below) with WMD −10.3 mmHg (95% CI −13.4, −7.1, *p* = 0.004, I^2^ 62.8%) and −5.3 mmHg (95% CI −7.5, −3.2, *p* = 0.021, I^2^ 53.8%), respectively. Furthermore, we also grouped the studies based on the position in which the blood pressure was measured. For both the supine and seated positions, WMD of systolic and diastolic BP was lower in tetraplegia compared to individuals with paraplegia and were in line with the overall findings. For systolic BP, the WMD were −12.4 mmHg (95% CI −28.4, −3.7, *p* < 0.001, I^2^ 93.0%) for supine and −13.2 mmHg (95% CI −18.8, −7.4, *p* < 0.001, I^2^ 88.3%) for seated, both lower in individuals with tetraplegia than paraplegia. For diastolic BP, the WMD were −6.6 mmHg (95% CI −12.9, −0.4, *p* = 0.023, I^2^ 68.4%) for supine and −6.4 mmHg (95% CI −9.6, −3.3, *p* < 0.001, I2 79.0%) for seated, which were both lower for individuals with tetraplegia than paraplegia. (Table 2).

**Table 2:** Summary of the effect estimates across different intermediate cardiovascular parameters.

Outcome, units	Number of studies	High injury, *N*^1^	High injury, mean (SD)	Low injury, *N*^2^	Low injury, mean (SD)	Weighted mean difference (95% CI)	*P* value for heterogeneity	I^2^ for heterogeneity
**Tetraplegia versus paraplegia**								
Blood pressure								
Systolic blood pressure, mmHg	20	733	114.6 (15.6)	783	125.1 (14.5)	−14.5 (−19.2, −9.9)	<0.001	92.5%
• Supine	4	67	114.9 (18.4)	80	123.1 (15.3)	−12.4 (−28.4, −3.7)	<0.001	93.0%
• Seated	11	384	112.6 (17.3)	477	125.2 (15.8)	−13.2 (−18.8, −7.4)	<0.001	88.3%
Diastolic blood pressure, mmHg	20	733	67.9 (11.8)	783	75.3 (9.4)	−7.0 (−9.2, −4.8)	<0.001	79.7%
• Supine	4	67	69.9 (12.2)	80	75.1 (10.6)	−6.6 (−12.9, −0.4)	0.023	68.4%
• Seated	11	384	69.0 (11.5)	477	76.1 (10.4)	−6.4 (−9.6, −3.3)	<0.001	79.0%
Mean arterial pressure, mmHg	5	68	80.2 (13.3)	723	91.3 (11.8)	−15.2 (−25.0, −5.4)	<0.001	87.3%
Lipid profile								
Triglycerides, mg/dL,	22	898	122.8 (58.9)	1052	131.7 (66.1)	−10.9 (−19.7, −2.1)	<0.001	93.2%
Total cholesterol, mg/dL	21	880	174 (29.9)	1036	186.2 (34.1)	−9.9 (−14.5, −5.4)	<0.001	91.5%
High density lipoprotein, mg/dL	22	899	40.5 (9.99)	1052	42.3 (9.9)	−1.7 (−3.3, −0.2)	<0.001	95.8%
Low density lipoprotein, mg/dL	20	836	108.6 (24.4)	988	117.3 (29.2)	−5.8 (−9.0, −2.5)	<0.001	82.8%
Cholesterol-HDL ratio	9	196	4.48 (1.17)	360	4.67 (1.43)	−0.22 (−0.53, 0.09)	0.005	63.7%
HDL-LDL ratio	4	165	3.23 (0.63)	209	3.18 (0.96)	−0.1 (−0.4, 0.3)	<0.001	96.7%
Glucose metabolism								
Glucose, mg/dL	13	433	95.9 (19.7)	552	93.4 (18.8)	−0.5 (−1.9, 1.0)	0.084	37.5%
Insulin, microUnits/mL	6	155	9.8 (5.4)	248	8.9 (4.2)	−0.3 (−1.8, 1.1)	<0.001	81.6%
HOMA IR^3^	4	88	2.1 (1.8)	126	1.7 (1.3)	0.1 (−0.41, 0.62)	0.128	56.7%
Inflammation markers								
C reactive protein, mg/L	2	36	1.78 (1.53)	40	1.53 (1.05)	0.20 (−0.23, 0.64)	0.888	0.0%
High-sensitivity C-reactive protein, mg/L	4	74	4.14 (3.91)	125	3.93 (3.95)	0.56 (−0.25, 1.37)	0.176	39.4%
Interleukin 6, pg/mL	4	79	4.50 (8.49)	120	5.29 (12.31)	−0.64 (−3.91, 2.62)	0.252	26.7%
Tumor necrosis factor alpha, pg/mL	3	59	51.10 (139.88)	78	57.39 (124.46)	0.22 (−6.75, 7.20)	0.361	1.8%
**High paraplegia (at T5 and above) versus low paraplegia (at T6 and below)**								
Blood pressure								
Systolic blood pressure, mmHg	10	466	121.3 (14.3)	295	129.7 (14.0)	−10.3 (−13.4, −7.1)	0.004	62.8%
• Seated	6	247	120.9 (17.2)	203	129.6 (15.4)	−9.8 (−15.1, −4.4)	0.002	72.0%
Diastolic blood pressure, mmHg	10	466	69.7 (12.6)	295	76.2 (9.7)	−5.3 (−7.5, −3.2)	0.021	53.8%
• Seated	6	245	72.7 (13.1)	213	76.7 (10.8)	−5.0 (−7.6, −2.4)	0.098	44.0%
Lipid profile								
Triglycerides, mg/dL	2	28	130.0 (55.5)	26	105.3 (40.0)	25.9 (14.9, 36.9)	0.935	0.0%
Total cholesterol, mg/dL	2	28	191.9 (32.3)	26	199.5 (39.0)	−12.6 (−43.1, 17.9)	0.083	66.6%
High density lipoprotein, mg/dL	2	28	43.5 (5.0)	26	52.2 (7.1)	−9.5 (−21.2, 2.1)	<0.001	92.7%
Low density lipoprotein, mg/dL	2	28	120.6 (26.5)	26	124 (38.2)	−10.0 (−25.5, 5.5)	0.245	26.1%
Cholesterol-HDL ratio	2	28	4.55 (1.16)	26	4.04 (1.18)	0.6 (0.2, 1.0)	0.791	0.0%

^1^High injury refers to either tetraplegia or high-paraplegia (at T5 and above) depending on the analysis subgroup.

^2^Low injury refers to paraplegia or low-paraplegia (at T6 and below) depending on the analysis subgroup.2Low injury refers to paraplegia or low-paraplegia (at T6 and below) depending on the analysis subgroup.

^3^HOMA IR homeostatic model assessment for insulin resistance (Matthews et al., 1985, Diabetologia, 28:412) 3HOMA IR homeostatic model assessment for insulin resistance (Matthews et al., 1985, Diabetologia, 28:412)

### Serum lipids

We analyzed 22 studies with 1951 individuals with SCI reporting differences in lipid profiles (Table 2). In general, serum lipids were lower in tetraplegia compared to paraplegia. Serum triglycerides and total cholesterol levels were lower in tetraplegia as compared to paraplegia with WMD −10.9 mg/dL (95% CI −19.7, −2.1, *p* < 0.001, I^2^ 93.2%) and −9.9 mg/dL (95% CI −14.5, −5.4, *p* < 0.001, I^2^ 91.5%), respectively. High-density lipoprotein (HDL) and low-density lipoprotein (LDL) was also lower in tetraplegia compared to paraplegia (WMD −1.7 mg/dL, 95% CI −3.3, −0.2, *p* < 0.001, I^2^ 95.8% and −5.8 mg/dL, 95% CI −9.0, −2.5 *p* < 0.001, I^2^ 82.8%). Results in the male-only population were in line with the overall findings (Appendix Table A3-a). We found no significant differences in HDL-LDL ratio and cholesterol-HDL ratio levels among the two groups.

### Glucose metabolism

Based on findings from 13 studies and 985 participants, mean glucose levels in individuals with tetraplegia was 95.9 mg/dL ± 19.7 compared with paraplegia with 93.4 mg/dL ± 18.8, but pooled estimates did not show a significant difference (WMD −0.5 mg/dL, 95% CI −1.9, 1.0, *p* = 0.084, I^2^ 37.5%) (Table 2). The mean serum insulin levels in individuals with tetraplegia were 9.8 uU/mL ± 5.4 compared to those with paraplegia 8.9 uU/mL ± 4.2, and the pooled difference between the groups was not statistically significant (WMD −0.3, 95% CI −1.8, 1.1, *p* < 0.001, I^2^ 81.6%). When comparing male-only WMD with mixed-population estimates, we did not find statistically significant differences in glucose and insulin levels between the two groups (tetraplegia and paraplegia) (Appendix Table A3-a).

### Inflammation and oxidative stress markers

Based on 4 studies and 199 individuals, the mean levels of high-sensitivity C-reactive protein (hsCRP) in tetraplegia was 4.14 mg/L ± 3.91 compared to 3.93 mg/L ± 3.95 for individuals with paraplegia. However, the WMD was not significantly different between the two groups (0.56 mg/L, 95% CI −0.25, 1.37, *p* = 0.176, I^2^ 39.4%). No differences were observed with interleukin 6 (4 studies, 199 individuals) and tumor necrosis factor-alpha (3 studies, 137 individuals) (Table 2) for different injury levels. Similarly, when comparing male-only WMD with mixed-population estimates, we did not find statistically significant differences in CRP levels between the two groups (tetraplegia and paraplegia) (Appendix Table A3-a).

### Sensitivity analyses and assessments of bias, study quality and heterogeneity

The quality of the majority (87%) of observational studies included in meta-analyses was judged to be of good quality (Newcastle-Ottawa Scale score ranged from 8 to 10) (Appendix Table A1). In general, meta-analyses showed high between-study heterogeneity, with an *I*^2^ estimate exceeding 75% (*p* < 0.05 for the Cochrane χ2 statistic) (Appendix Fig. A1). The heterogeneity of the outcomes was barely explained by the meta-regression analysis. However, factors such as age, body mass index (BMI), prevalent CVD, lesion duration, sample size, and study quality are suggested to be the sources of heterogeneity across different outcomes (Appendix Table A3a & b). BMI was seen as source of heterogeneity for HDL. Baseline cardiovascular disease and the use of cardiovascular medications were also documented. However, except for an indication that this may be a source of heterogeneity in meta-analysis focusing on triglycerides and HDL, no significant differences were observed across the risk factors. We also used meta-regression to fit a regression line on patient characteristics and the cardiovascular risk factors (Appendix Table A4). BMI seemed to affect the disparity of insulin between individuals with tetraplegia and paraplegia. The disparity in insulin increased with increasing BMI (β 0.94, 95% CI 0.29, 1.60). Age influenced HDL (β 0.18, 95% CI 0.01, 0.35) Duration of injury also influenced HDL and insulin levels, as both increase together with longer duration of injury (β 0.37, 95% CI 0.08, 0.66, and 1.02, 95% CI 0.07, 1.98, respectively) (Appendix Table A4).

Leave-one-out analyses were done to determine if one study was driving the overall effect estimate (Appendix Fig. A2). The removal of the study of Wang et al. [20] changed the pooled estimate, so that individuals with tetraplegia had higher insulin levels compared to paraplegia (WMD 0.74 uU/mL, 95% CI 0.24, 1.23). This study included those with infections which was not done in other studies assessing insulin levels. By removing the study of O’Brien, et al. [21], the estimates were also changed with tetraplegia having a lower cholesterol-HDL ratio compared to paraplegia (WMD −0.5, 95% CI −0.64, −0.35). Tests for publication bias showed mostly symmetric distribution in funnel plots (Appendix Fig. A3), except for HOMA-IR (Egger’s test *p* = 0.019), cholesterol HDL ratio (Egger’s test *p* = 0.025), and HDL (Egger’s test *p* = 0.102).

## Discussion

**Fig. 2:**
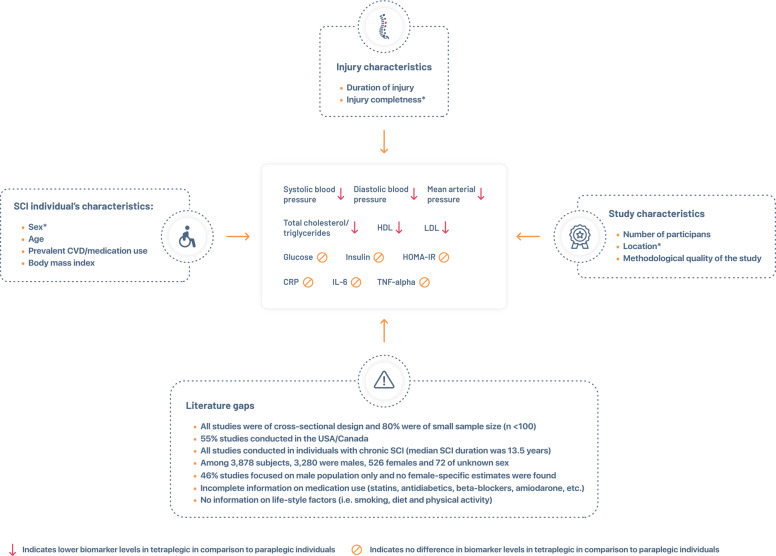
Illustrative summary of the most important findings and literature gaps. (*in meta-regression analysis this factor was not indicated as a source of heterogeneity; HDL, high density lipoprotein; LDL, low density lipoprotein; CRP, c-reactive protein; IL-6, interleukin 6; TNF-alpha, tumor necrosis factor alpha; HOMA-IR, homeostatic model assessment for insulin resistance).

In this systematic review, a higher injury level (tetraplegia) was associated with lower BP (systolic BP, diastolic BP, mean arterial pressure MAP) compared to a lower injury level (paraplegia). A higher injury level was associated with lower HDL in lipid profile, albeit with lower triglycerides, total cholesterol, and LDL. We found no differences in glucose metabolism and inflammation markers. As some literature suggests higher CVD incidence in SCI individuals with tetraplegia compared to paraplegia, our study determined an exact difference between the two groups according to their modifiable risk factors (eg., blood pressure and serum lipids). Our results, however, were based mostly on relatively young individuals, male population, chronic SCI, and studies conducted in Europe and North America. Thus, our findings may not be generalizable to all individuals with SCI. An illustrative summary of the most critical findings and literature gaps is provided in Fig. 2.

To the best of our knowledge, this is the first systematic review that specifically focuses on the level of injury (as well as its completeness and duration) and CVD risk factors. We identified two systematic reviews which primarily compared the BP and blood lipids between individuals with SCI and ABI. Their respective subgroup analyses, comparing across injury levels, were similar to our findings. In the meta-analysis of West et al. including 21 studies, authors reported lower BP and heart rate values in SCI individuals with a higher (cervical) compared to lower injury level (high thoracic T1-T5 and low thoracic T6 below) [17]. In another meta-analysis by Gilbert et al., total cholesterol and non-HDL cholesterol were significantly lower in the tetraplegia subgroup, while LDL and triglycerides were not different among individuals with tetraplegia and paraplegia [16]. Our review included more studies, contains more up-to-date articles, provided male-specific analyses, and included more cardiovascular risk factors. In addition, we used newer meta-analysis methods such as stratification analysis and meta-regression to explore the role of other factors that may mediate the association between injury level and CVD risk factors such as individual study (e.g., number of study participants, study location) and study participants characteristics (e.g., percentage of males, age) and characteristics of the injury (completeness and injury duration).

In SCI, the impairment ranges from the motor to neurohormonal control below the level of injury. The autonomics play a crucial role in the neurologic and hormonal control of the vascular tone. It also plays a role in the homeostatic control of fluids, electrolytes, glucose, and lipid metabolism [22]. The motor system is crucial in controlling the movement of limbs and the spine (appendicular and axial muscles), which determines an individual’s mobility and physical activity. Thus, there could be a lesion-level-dependent difference in CVD risk in the SCI population mediated via altered autonomic function, metabolism, and physical activity levels [23]. Indeed, in a retrospective cohort study, the risk of clinically apparent CVD was the highest in individuals with tetraplegia and individuals with complete SCI as compared to other injury modalities [24]. Similarly, atherosclerosis burden was different according to injury level, carotid intima media thickness was higher in tetraplegic subjects in comparison to paraplegic ones, and individuals with tetraplegia and complete SCI had the highest prevalence of silent coronary artery disease and a greater prevalence of severe coronary artery calcification scores than those with paraplegia [25–27].

Here we discuss potential mechanisms underlying the differences in cardiovascular risk factors observed in current systematic reviews. The involvement of the autonomic nervous system was suggested as the major mechanism in the blood pressure differences across different injury levels. The autonomics play a crucial function in the neurologic and hormonal control of the vascular tone and can also play a role in the endocrine control of fluids and electrolytes critical to BP control. In particular, autonomic dysreflexia (usually present in individuals with damage at or above T6 spinal segment) may mediate increased CVD risk in individuals with tetraplegia compared to paraplegia via structural and functional modifications to peripheral and cerebral vasculatures (due to repeated episodes of vascular contractions and associated hypertension which occasionally can reach extreme levels) [28]. Thus, considering that in the current study we were not able to control for the diagnosis of autonomic dysreflexia, our findings on lower systolic and diastolic BP and MAP observed in tetraplegic individuals should be interpreted with caution (e.g., in individuals with SCI, the association between the BP values and long-term cardiovascular disease risk may not follow the same trend as compared to the ABI).

In addition to lower HDL in individuals with tetraplegia as compared to paraplegia, we reported lower total cholesterol, LDL, and triglycerides levels in the former group. In the subgroup analyses exploring the lipid levels according to the different levels of injury, two reviews had contradictory findings. Wilt et al. did not find any significant difference [12], while Gilbert et al. reported lower cholesterol and HDL in individuals with tetraplegia [16]. Exercise or involvement in physical activity may increase HDL levels and improve its antioxidative and anti-inflammatory properties, which may explain higher levels in individuals with paraplegia who are able to exercise with full use of upper limbs and the trunk, and thus are more physically active as compared to tetraplegic individuals [29]. In addition, the majority of studies did not provide information on statin use (which are mainly prescribed due to their LDL-lowering properties), and therefore, we cannot speculate whether lower total cholesterol, triglycerides, and LDL levels seen in individuals with tetraplegia may be affected by perhaps higher prevalence of statin-use in this group. Also, some authors suggest that the lipid ratios (HDL-LDL ratio and cholesterol-HDL) ratio may be a more important determinant of increased CVD risk in individuals with SCI considering all the physiological changes after injury [16]. In previous systematic review, they observed that individuals with SCI have a higher cholesterol HDL ratio than ABI, thus, the authors speculated that the former have poorer cardiovascular profile than the latter [16]. In our review, in six out of nine studies we observed higher cholesterol-HDL ratio in tetraplegia (poorer lipid profile). Still, pooled estimates did not show statistically significant differences, which might be attributed to limited number of studies included in analyses or the heterogeneity among available studies.

Furthermore, we found no differences between the two groups in glucose metabolism and inflammation parameters. The determinants of glucose metabolism have been proven to be more complex. In a large study on glucose metabolism, individuals with tetraplegia had poorer glucose and insulin profiles than paraplegia (after oral glucose tolerance test) [30]. In contrast, studies comparing fasting blood glucose according to injury level reported no differences [20, 31, 32]. Conflicting findings could be a consequence of methodological differences between the studies and the fact that fasting blood glucose may be less sensitive than oral glucose tolerance tests in detecting hyperglycemia. Also, other mitigating factors such as baseline inflammation [33] and physical activity [34] may play important roles in glucose control, however, studies have only compared the mean differences between the groups without considering these confounding factors. In fact, there is a general trend of higher inflammation markers in SCI individuals, which is a consequence of chronic urinary tract infection, skin ulcers, and reduced physical activity, among others [20, 35, 36]. Limited evidence also suggests higher inflammation status in tetraplegia compared to paraplegia. However, the studies had small sample sizes, and several confounding factors were not considered. As such, the influence of the level of injury is still poorly described. Similar to glucose metabolism, the complex interaction of neurohormonal control and physical activity through the motor pathway could determine the inflammation marker levels.

In addition to the injury level, the completeness of the injury and its duration are also important contributing factors that can affect cardiovascular risk factors. As seen in our subgroup analyses, the completeness and injury duration were additional injury-related factors that could possibly mediate the CVD risk but needs further research to be proven. Completeness of injury is usually assessed using the ASIA impairment scale. This scale, however, fails to consider the autonomic function. And as such, reviews have not fully elucidated its influence on blood pressure and lipids. As for the duration of injury, factor analysis has been challenging as age is highly correlated with injury duration (36, 37). A longitudinal study has shown that quality of life, activity, and participation declines with the duration of injury, along with age and age of onset (38).

### Strengths and limitations

Studies in SCI frequently lack statistical power due to small sample sizes. This is primarily due to the low incidence of SCI, few registries that collect standardized information on SCI, and few collaborative studies across different rehabilitation centers [37]. Therefore, combining studies using meta-analysis may solve this issue. To our knowledge, this is the largest meta-analysis focusing on the level of SCI and cardiovascular disease risk factors. To identify as many relevant studies as possible and reduce the risk of publication bias, a highly sensitive search strategy was used, and additional resources were searched, including the reference list of included studies and relevant systematic reviews. We also included studies that did not include CVD as a primary outcome but provided information on CVD risk factors to include more individuals in our pooled estimate.

However, we report some weaknesses that need to be considered when interpreting our data. First, although the studies included were of good quality, these were observational studies and provided unadjusted mean biomarker levels, precluding our ability to speculate on causality. Second, there were crucial gaps in the reporting of other critical cardiovascular risk factors in most studies. Most studies did not report smoking history and medication use. Physical activity, diet, and other lifestyle factors were also seldomly reported. Third, we did not identify any study exploring the association between injury level and markers of endothelial/vascular function and carotid atherosclerosis, which would have strengthened our findings. Fourth, some studies have used similar study participants, which may result in double reporting. We solved this problem by comparing the study characteristics and including the latest publication only or the study with the most relevant outcome of interest. Finally, females were grossly underrepresented in our analysis (526/3878 or 13.5%), thus, our analysis could only be generalized to males. Despite sex is a  critical determinant of cardiovascular risk, the SCI researches rarely included females or performed female-specific analyses and we discussed this phenomenon elsewhere [38].

### Research gaps and implications for future research

Although SCI individuals are considered to have increased cardiovascular risk, the number of SCI-specific clinical guidelines is limited, and validated CVD risk prediction models for SCI are lacking [39]. Understanding how the injury contributes to the overall cardiovascular risk in SCI individuals is crucial in tailoring the preventive strategies in this high-risk population. The risk prediction models used in ABI (e.g., FRS) may not be valid estimators of future cardiovascular events in SCI individuals. The results of the current meta-analysis suggest that the anatomic level of the injury may be a critical non-modifiable CVD risk factor for this population. Sufficiently powered longitudinal studies should be done to establish causality. Future studies should also include individuals in subacute SCI and include both males and females to allow sex-specific analyses. These subsequent studies would improve our understanding of the role of the injury level in modifying CVD risk and whether the injury level may be used for risk stratification and development of injury-specific prevention and treatment strategies.

## Conclusion

Our findings suggest that the injury level may be an additional non-modifiable cardiovascular risk factor for individuals with SCI. In SCI individuals with a higher injury level, blood pressure was lower compared to lower injury. In regards to lipids, individuals with higher injury levels have lower HDL, albeit with lower cholesterol, triglycerides, and LDL. We found no differences in glucose metabolism and inflammation markers. Factors such as age, prevalent CVD and medication use, BMI, sample size, and quality of studies may be important factors that affect our estimates and could have precluded our ability to detect differences in glucose metabolism parameters. To better understand the role of injury level in modifying CVD risk and confirm our findings, future longitudinal studies should be sufficiently powered and include individuals with subacute and chronic SCI, including both males and females, to allow for sex-specific analysis.

## Supplementary information

Online supplement
