# Supplementary material for: The neurological level of spinal cord injury and cardiovascular risk factors: a systematic review and meta-analysis
Source: Spinal Cord. 2021 Aug 20;59(11):1135–45. doi: 10.1038/s41393-021-00678-6 (PMC8560636; doi:10.1038/s41393-021-00678-6)
Supplement: Supplementary file 1 — Online supplement [file 41393_2021_678_MOESM1_ESM.docx]

**Search Strategy**

Is there an association between the neurologic level of spinal cord injury and the intermediate cardiometabolic risk factors, incident cardiometabolic diseases, and all-cause and CVD mortality?

July 4, 2020 (last date searched)

|  | Before deduplication | After deduplication |
| --- | --- | --- |
| Embase.com | 3802 | 3777 |
| Medline Ovid | 1597 | 412 |
| Cochrane CENTRAL | 299 | 180 |
| Web-of-Science | 1313 | 441 |
| Google Scholar | 400 | 53 |
| Total | 7411 | **4863** |

2548 duplicate records have been removed

**Embase.com**

('spinal cord injury'/exp OR 'cervical spine injury'/de OR 'spinal cord ischemia'/de OR 'paraplegia'/de OR 'spastic paraplegia'/de OR 'quadriplegia'/de OR 'spinal dysraphism'/de OR ('injury'/exp AND 'spinal cord'/exp) OR (((spine or spinal) NEAR/3 (injur* or trauma* or damag*)) OR ('spinal cord' NEAR/3 (disease* or contusion* or laceration* or transection* or lesion* or trauma* or ischemi* or ischaemi*)) OR (myelopath* NEAR/3 (trauma* or post-trauma* or posttrauma*)) OR ((spine or spinal or vertebra*) NEAR/3 (fracture* or trauma* or injur* or damage* or wound*)) OR 'central cord injury syndrome*' OR 'central cord syndrome*' OR 'central spinal cord syndrome*' OR 'cauda equine syndrome*' OR 'anterior cord syndrome*' OR 'conus medullaris syndrome*' OR 'Brown Sequard' OR paraplegi* OR quadriplegi* OR tetraplegi*):ab,ti)

AND

('disease severity'/de OR 'injury severity'/de OR 'severity of illness index'/exp OR 'disease marker'/de OR (((level* OR severit* OR completeness OR degree* OR marker*) NEAR/6 (injur* OR lesion* OR illness* OR disease* OR SCI)) OR (neurologic* NEAR/1 level*) OR (lesion* NEAR/3 dependen*)):ab,ti)

AND

('diabetes mellitus'/exp OR 'cardiovascular disease'/de OR 'heart failure'/de OR 'congestive heart failure'/de OR 'heart disease'/de OR 'cardiovascular risk'/exp OR 'cardiometabolic risk'/de OR 'cardiovascular mortality'/de OR 'blood pressure'/exp OR 'hypertension'/de OR 'coronary artery disease'/de OR 'ischemic heart disease'/exp OR 'cerebrovascular accident'/de OR 'venous thromboembolism'/de OR 'atherosclerotic cardiovascular disease'/de OR 'brain ischemia'/exp OR 'insulin response'/exp OR 'glucose blood level'/exp OR 'insulin blood level'/exp OR 'hyperinsulinism'/exp OR 'lipid blood level'/exp OR 'lipid level'/exp OR 'glycosylated hemoglobin'/exp OR 'C reactive protein'/de OR 'c reactive protein blood level'/de OR (inflammation/de AND (marker/de OR 'C reactive protein'/exp OR cytokine/de OR fibrinolysis/exp OR 'tumor necrosis factor alpha'/exp)) OR 'chronic inflammation'/exp OR atherosclerosis/de OR 'atherosclerotic plaque'/de OR 'carotid atherosclerosis'/exp OR 'coronary artery atherosclerosis'/exp OR 'obesity'/de OR 'body mass'/de OR 'abdominal obesity'/de OR 'waist circumference'/de OR 'metabolic disorder'/exp OR 'oxidative stress'/de OR 'reactive oxygen metabolite'/de OR 'lipid peroxidation'/de OR 'isoprostane derivative'/de OR 'malonaldehyde'/de OR 'lipoxygenase'/de OR 'myeloperoxidase'/de OR 'endothelium derived relaxing factor'/de OR 'nitric oxide'/de OR 'prostacyclin derivative'/exp OR 'endothelium derived hyperpolarizing factor'/de OR 'endothelium derived constricting factor'/de OR 'endothelin 1'/de OR 'intercellular adhesion molecule 1'/de OR 'vascular cell adhesion molecule 1'/de OR 'endothelial leukocyte adhesion molecule 1'/de OR 'PADGEM protein'/de OR 'arterial stiffness'/de OR 'heart function'/exp OR 'cardiovascular function'/exp OR 'flow-mediated dilation test'/de OR 'peripheral arterial tonometry'/de OR 'heart stroke volume'/de OR (diabet* OR HbA1c OR HbA-1c OR ((cardiovascular OR coronar* OR cardiac OR heart OR cardiometabol* OR cardio-metabol* OR metabolic) NEAR/3 (disease* OR event* OR disorder* OR syndrome* OR function* OR dysfunction* OR health OR mortality OR risk*)) OR cvd OR cvds OR CV-risk OR 'blood pressure*' OR hypertension OR ((ischemi* OR ischaemi* OR fail* OR insufficien* OR infarct*) NEAR/3 (heart OR cardia* OR myocard*)) OR (cerebrovascular* NEAR/3 accident*) OR cva OR stroke* OR cardiopath* OR angina OR ((brain OR cerebral) NEAR/3 (ischemi* OR ischaemi*)) OR ((glucose OR sugar OR insulin* OR lipid* OR cholester* OR lipoprotein* OR triacylglycerol* OR triglyceride*) NEAR/6 (level* OR blood OR serum OR plasma* OR concentration*)) OR dyslipidemia* OR dyslipidaemia* OR glucosaem* OR glucosem* OR glycaem* OR glycem* OR hyperinsulin* OR hypoinsulin* OR insulinaem* OR insulinem* OR (insulin NEAR/3 (response OR dependen* OR resistan* OR sensitiv*)) OR hypercholesterol* OR (inflammat* NEAR/3 (chronic* OR marker* OR biomarker* OR interleukin* OR crp OR 'c reactive' OR cytokine* OR leptin* OR fibrinolys* OR fibrinogenlys* OR 'tumor necrosis factor' OR tnf)) OR atheroscler* OR arterioscler* OR homocysteine* OR obes* OR adipos* OR (waist NEAR/3 (circumference* or hip)) OR 'body mass' OR 'body size' OR 'body weight' OR bmi OR (oxidative NEAR/3 stress*) OR (reactive NEAR/3 oxygen* NEAR/3 (metabolite* OR species)) OR (lipid* NEAR/3 (peroxidat* OR autooxidat* OR autoxidat*)) OR lipoperoxidat* OR lipo-peroxidat* OR isoprostan* OR malonaldehyde* OR lipoxygenase* OR myeloperoxidase* OR 'endothelial-derived relaxing factor' OR 'nitric oxide' OR prostaglandin-i2 OR PGI2 OR 'endothelium derived hyperpolarising factor*' OR 'endothelium derived constricting factor*' OR 'endothelium derived contracting factor*' OR endothelin-1 OR 'intercellular adhesion molecule-1' OR 'vascular cell adhesion molecule-1' OR E-selectin OR selectin-E OR P-selectin OR selectin-P OR ((vascular OR arterial) NEAR/1 (stiffness)) OR ((arterial) NEAR/1 (wall OR thickness)) OR ((ventricular OR ventricle OR heart) NEAR/3 (function* OR dysfunction* OR rate*)) OR 'ventricle stroke volume' OR 'pulse wave' OR 'flow-mediated dilatation' OR 'peripheral arterial tonometry' OR echocardiograph*):ab,ti)

NOT ([animals]/lim NOT [humans]/lim) NOT ([Conference Abstract]/lim OR [Letter]/lim OR [Note]/lim OR [Editorial]/lim)

**Medline Ovid**

(exp Spinal Cord Injuries/ OR exp Spinal Cord Ischemia/ OR exp Paraplegia/ OR Quadriplegia/ OR Spinal Dysraphism/ OR (((spine or spinal) ADJ3 (injur* or trauma* or damag*)) OR (spinal cord ADJ3 (disease* or contusion* or laceration* or transection* or lesion* or trauma* or ischemi* or ischaemi*)) OR (myelopath* ADJ3 (trauma* or post-trauma* or posttrauma*)) OR ((spine or spinal or vertebra*) ADJ3 (fracture* or trauma* or injur* or damage* or wound*)) OR central cord injury syndrome* OR central cord syndrome* OR central spinal cord syndrome* OR cauda equine syndrome* OR anterior cord syndrome* OR conus medullaris syndrome* OR Brown Sequard OR paraplegi* or quadriplegi* or tetraplegi*).ab,ti.)

AND

("Severity of Illness Index"/ OR (((level* OR severit* OR completeness OR degree* OR marker*) ADJ6 (injur* OR lesion* OR illness* OR disease* OR SCI)) OR (neurologic* ADJ1 level*) OR (lesion* ADJ3 dependen*)).ab,ti.)

AND

(exp "Diabetes Mellitus"/ OR "Cardiovascular Diseases"/ OR "Heart Failure"/ OR "Heart Diseases"/ OR exp Blood Pressure/ OR exp "Coronary Artery Disease"/ OR exp "Myocardial Ischemia"/ OR exp "Stroke"/ OR Venous Thromboembolism/ OR Hypertension/ OR "Atherosclerosis"/ OR exp "Brain Ischemia"/ OR "Insulin Resistance"/ OR glucose/bl OR insulin/bl OR exp Hyperinsulinism/ OR lipids/bl OR Glycated Hemoglobin A/ OR C-Reactive Protein/OR (inflammation/ AND (biomarkers/ OR "C-Reactive Protein"/ OR cytokines/ OR fibrinolysis/ OR "Tumor Necrosis Factor-alpha"/)) OR Atherosclerosis/ OR "Plaque, Atherosclerotic"/ OR "Carotid Artery Diseases"/ OR exp obesity/ OR "Body Mass Index"/ OR Waist Circumference/ OR Metabolic Diseases/ OR Oxidative Stress/ OR Reactive Oxygen Species/ OR Lipid Peroxidation/ OR Isoprostanes derivative/ OR Malondialdehyde/ OR Lipoxygenase/ OR Peroxidase/ OR Endothelium-Dependent Relaxing Factors/ OR Nitric Oxide/ OR exp prostaglandins i/ OR Endothelin-1/ OR Nitrites/ OR Cell Adhesion Molecules/ OR Intercellular Adhesion Molecule-1/ OR Vascular Cell Adhesion Molecule-1/ OR e-selectin/ OR p-selectin/ OR Vascular Stiffness/ OR Pulse Wave Analysis/ OR exp Heart Function Tests/ OR Ventricular Dysfunction/ OR (diabet* OR HbA1c OR HbA-1c OR ((cardiovascular OR coronar* OR cardiac OR heart OR cardiometabol* OR cardio-metabol* OR metabolic) ADJ3 (disease* OR event* OR disorder* OR syndrome* OR function* OR dysfunction* OR health OR mortality OR risk*)) OR cvd OR cvds OR CV-risk OR blood pressure* OR ((ischemi* OR ischaemi* OR fail* OR insufficien* OR infarct*) ADJ3 (heart OR cardia* OR myocard*)) OR (cerebrovascular* ADJ3 accident*) OR cva OR stroke* OR cardiopath* OR angina OR ((brain OR cerebral) ADJ3 (ischemi* OR ischaemi*)) OR ((glucose OR sugar OR insulin* OR lipid* OR cholester* OR lipoprotein* OR triacylglycerol* OR triglyceride*) ADJ6 (level* OR blood OR serum OR plasma* OR concentration*)) OR dyslipidemia* OR dyslipidaemia* OR glucosaem* OR glucosem* OR glycaem* OR glycem* OR hyperinsulin* OR hypoinsulin* OR insulinaem* OR insulinem* OR (insulin ADJ3 (response OR dependen* OR resistan* OR sensitiv*)) OR hypercholesterol* OR (inflammat* ADJ3 (chronic* OR marker* OR biomarker* OR interleukin* OR crp OR c reactive OR cytokine* OR leptin* OR fibrinolys* OR fibrinogenlys* OR tumor necrosis factor OR tnf)) OR atheroscler* OR arterioscler* OR homocysteine* OR obes* OR adipos* OR (waist ADJ3 (circumference* or hip)) OR body mass OR body size OR body weight OR bmi OR (oxidative ADJ3 stress*) OR (reactive ADJ3 oxygen* ADJ3 (metabolite* OR species)) OR (lipid* ADJ3 (peroxidat* OR autooxidat* OR autoxidat*)) OR lipoperoxidat* OR lipo-peroxidat* OR isoprostan* OR malonaldehyde* OR lipoxygenase* OR myeloperoxidase* OR endothelial-derived relaxing factor OR nitric oxide OR prostaglandin-i2 OR PGI2 OR endothelium derived hyperpolarising factor* OR endothelium derived constricting factor* OR endothelium derived contracting factor* OR endothelin-1 OR intercellular adhesion molecule-1 OR vascular cell adhesion molecule-1 OR E-selectin OR selectin-E OR P-selectin OR selectin-P OR ((vascular OR arterial) ADJ1 (stiffness)) OR ((arterial) ADJ1 (wall OR thickness)) OR ((ventricular OR ventricle OR heart) ADJ3 (function OR dysfunction OR rate)) OR ventricle stroke volume OR pulse wave OR flow-mediated dilatation OR peripheral arterial tonometry OR echocardiograph*).ab,ti.)

NOT (exp animals/ NOT humans/) NOT (letter OR news OR comment OR editorial OR congresses OR abstracts).pt.

**Cochrane CENTRAL: Cochrane Central Register of Controlled Trials, Issue 7 of 12, July 2020**

((((spine or spinal) NEAR/3 (injur* or trauma* or damag*)) OR ("spinal cord" NEAR/3 (disease* or contusion* or laceration* or transection* or lesion* or trauma* or ischemi* or ischaemi*)) OR (myelopath* NEAR/3 (trauma* or post-trauma* or posttrauma*)) OR ((spine or spinal or vertebra*) NEAR/3 (fracture* or trauma* or injur* or damage* or wound*)) OR "central cord injury syndrome" OR "central cord syndrome" OR "central spinal cord syndrome" OR "cauda equine syndrome" OR "anterior cord syndrome" OR "conus medullaris syndrome" OR "Brown Sequard" OR paraplegi* OR quadriplegi* OR tetraplegi*)):ab,ti,kw

AND

((((level* OR severit* OR completeness OR degree* OR marker*) NEAR/6 (injur* OR lesion* OR illness* OR disease* OR SCI)) OR (neurologic* NEAR/1 level*) OR (lesion* NEAR/3 dependen*))):ab,ti,kw

AND

((diabet* OR HbA1c OR HbA-1c OR ((cardiovascular OR coronar* OR cardiac OR heart OR cardiometabol* OR cardio-metabol* OR metabolic) NEAR/3 (disease* OR event* OR disorder* OR syndrome* OR function* OR dysfunction* OR health OR mortality OR risk*)) OR cvd OR cvds OR CV-risk OR blood-pressure* OR hypertension OR ((ischemi* OR ischaemi* OR fail* OR insufficien* OR infarct*) NEAR/3 (heart OR cardia* OR myocard*)) OR (cerebrovascular* NEAR/3 accident*) OR cva OR stroke* OR cardiopath* OR angina OR ((brain OR cerebral) NEAR/3 (ischemi* OR ischaemi*)) OR ((glucose OR sugar OR insulin* OR lipid* OR cholester* OR lipoprotein* OR triacylglycerol* OR triglyceride*) NEAR/6 (level* OR blood OR serum OR plasma* OR concentration*)) OR dyslipidemia* OR dyslipidaemia* OR glucosaem* OR glucosem* OR glycaem* OR glycem* OR hyperinsulin* OR hypoinsulin* OR insulinaem* OR insulinem* OR (insulin NEAR/3 (response OR dependen* OR resistan* OR sensitiv*)) OR hypercholesterol* OR (inflammat* NEAR/3 (chronic* OR marker* OR biomarker* OR interleukin* OR crp OR c-reactive OR cytokine* OR leptin* OR fibrinolys* OR fibrinogenlys* OR "tumor necrosis factor" OR tnf)) OR atheroscler* OR arterioscler* OR homocysteine* OR obes* OR adipos* OR (waist NEAR/3 (circumference* or hip)) OR "body mass" OR "body size" OR "body weight" OR bmi OR (oxidative NEAR/3 stress*) OR (reactive NEAR/3 oxygen* NEAR/3 (metabolite* OR species)) OR (lipid* NEAR/3 (peroxidat* OR autooxidat* OR autoxidat*)) OR lipoperoxidat* OR lipo-peroxidat* OR isoprostan* OR malonaldehyde* OR lipoxygenase* OR myeloperoxidase* OR "endothelial-derived relaxing factor" OR "nitric oxide" OR prostaglandin-i2 OR PGI2 OR endothelium-derived-hyperpolarising-factor* OR endothelium-derived-constricting-factor* OR endothelium-derived-contracting-factor* OR endothelin-1 OR "intercellular adhesion molecule-1" OR "vascular cell adhesion molecule-1" OR E-selectin OR selectin-E OR P-selectin OR selectin-P OR ((vascular OR arterial) NEAR/1 (stiffness)) OR ((arterial) NEAR/1 (wall OR thickness)) OR ((ventricular OR ventricle OR heart) NEAR/3 (function* OR dysfunction* OR rate*)) OR "ventricle stroke volume" OR "pulse wave" OR "flow-mediated dilatation" OR "peripheral arterial tonometry" OR echocardiograph*)):ab,ti,kw

Web of Science

TS=((((spine or spinal) NEAR/3 (injur* or trauma* or damag*)) OR ("spinal cord" NEAR/3 (disease* or contusion* or laceration* or transection* or lesion* or trauma* or ischemi* or ischaemi*)) OR (myelopath* NEAR/3 (trauma* or post-trauma* or posttrauma*)) OR ((spine or spinal or vertebra*) NEAR/3 (fracture* or trauma* or injur* or damage* or wound*)) OR "central cord injury syndrome" OR "central cord syndrome" OR "central spinal cord syndrome" OR "cauda equine syndrome" OR "anterior cord syndrome" OR "conus medullaris syndrome" OR "Brown Sequard" OR paraplegi* OR quadriplegi* OR tetraplegi*))

AND

TS=((((level* OR severit* OR completeness OR degree* OR marker*) NEAR/6 (injur* OR lesion* OR illness* OR disease* OR SCI)) OR (neurologic* NEAR/1 level*) OR (lesion* NEAR/3 dependen*)))

AND

TS=((diabet* OR HbA1c OR HbA-1c OR ((cardiovascular OR coronar* OR cardiac OR heart OR cardiometabol* OR cardio-metabol* OR metabolic) NEAR/3 (disease* OR event* OR disorder* OR syndrome* OR function* OR dysfunction* OR health OR mortality OR risk*)) OR cvd OR cvds OR CV-risk OR "blood pressure*" OR hypertension OR ((ischemi* OR ischaemi* OR fail* OR insufficien* OR infarct*) NEAR/3 (heart OR cardia* OR myocard*)) OR (cerebrovascular* NEAR/3 accident*) OR cva OR stroke* OR cardiopath* OR angina OR ((brain OR cerebral) NEAR/3 (ischemi* OR ischaemi*)) OR ((glucose OR sugar OR insulin* OR lipid* OR cholester* OR lipoprotein* OR triacylglycerol* OR triglyceride*) NEAR/6 (level* OR blood OR serum OR plasma* OR concentration*)) OR dyslipidemia* OR dyslipidaemia* OR glucosaem* OR glucosem* OR glycaem* OR glycem* OR hyperinsulin* OR hypoinsulin* OR insulinaem* OR insulinem* OR (insulin NEAR/3 (response OR dependen* OR resistan* OR sensitiv*)) OR hypercholesterol* OR (inflammat* NEAR/3 (chronic* OR marker* OR biomarker* OR interleukin* OR crp OR c-reactive OR cytokine* OR leptin* OR fibrinolys* OR fibrinogenlys* OR "tumor necrosis factor*" OR tnf)) OR atheroscler* OR arterioscler* OR homocysteine* OR obes* OR adipos* OR (waist NEAR/3 (circumference* or hip)) OR "body mass" OR "body size" OR "body weight" OR bmi OR (oxidative NEAR/3 stress*) OR (reactive NEAR/3 oxygen* NEAR/3 (metabolite* OR species)) OR (lipid* NEAR/3 (peroxidat* OR autooxidat* OR autoxidat*)) OR lipoperoxidat* OR lipo-peroxidat* OR isoprostan* OR malonaldehyde* OR lipoxygenase* OR myeloperoxidase* OR "endothelial-derived relaxing factor" OR "nitric oxide" OR prostaglandin-i2 OR PGI2 OR "endothelium-derived hyperpolarizing factor*" OR "endothelium-derived constricting factor*" OR "endothelium-derived contracting factor*" OR endothelin-1 OR "intercellular adhesion molecule-1" OR "vascular cell adhesion molecule-1" OR E-selectin OR selectin-E OR P-selectin OR selectin-P OR ((vascular OR arterial) NEAR/1 (stiffness)) OR ((arterial) NEAR/1 (wall OR thickness)) OR ((ventricular OR ventricle OR heart) NEAR/3 (function* OR dysfunction* OR rate*)) OR "ventricle stroke volume*" OR "pulse wave*" OR "flow-mediated dilatation*" OR "peripheral arterial tonometry" OR echocardiograph*))

NOT TS=(((animal* OR rat OR rats OR mouse OR mice OR murine OR nonhuman* OR primate*) NOT (human* OR patient*)))

AND DT=(article)

**Google scholar**

*15900 results (14.07.2020)  first 400 according to relevance ranking (for the Update search: first 100 since 2016)*

Spinal Cord|SCI "level|levels of injury"|"lesion level"|"level of lesions"|"injury level|levels" diabetes|"cardiovascular|coronary|metabolic|cardiometabolic disease|events|syndromes|risk|mortality"|stroke|obesity|"body mass"|endothelial|"pulse wave"|vascular stiffness

Table A1. Characteristics of studies

| **Author, year of publication** | **Study Design** | **Study Location (country)** | **Population (setting)** | **No. of individuals** | **Sex, number (% male)** | **SCI lesion duration (mean y±SD )** | **Age (mean y±SD )** | **Classification of injury** | **Complete vs incomplete** | **Baseline cardiovascular status (disease/meds)** | **Outcomes** | **Study Quality** |
| --- | --- | --- | --- | --- | --- | --- | --- | --- | --- | --- | --- | --- |
| Aadriansen 2016 (1) | CS | Netherlands | 8 rehabilitation centers, ALLRISC consortium (patients within 10 years) | 282 | 209 (74.1) | 23.2 (3.34) | 47.8 (41.6–55.0) | TP C2-C8  PP-H T1-7  PP-L >T7 | 68.7% | HTN 19.2%/ drugs 6.7%; DM 7.1% | Blood pressure | 9 |
| Akbal 2013 (2) | CS | Turkey | Ankara Rehabilitation Research hospital, Jan 2008 to Aug 2009, (no cardiovascular disease baseline) | 56 | n.d. | 3.05 (2.84) | 32.1 (11.8) | TP vs PP | 13% | No CAD in the past | Blood pressure, lipids, glucose | 9 |
| Apstein 1998 (3) | CH | US | VA Medical Center, US | 100 | 100 (100) | *1 yr or 52 weeks | 45 (no s.d.) | TP vs PP | n.d. | n.d. | Lipids | 9 |
| Baumann 1999 (4) | CS | US | Los Amigos Medical Center, May 1994-May 1998 | 201 | 169 (84.1) | 13 (0.7) | 39 (0.8) | TP vs PP | 100% | n.d. | Lipids | 8 |
| Baumann 1992 (5) | CS | US | Veterans Affairs Medical Center | 100 | 100 (100) | 16.3 (1.7) | 47.9 (1.95) | TP vs PP | n.d. | n.d. | Lipids, | 7 |
| Bernardi 2019 (6) | CS | Italy | Pre-participation medical records prior to London 2012 and Sochi 2014, 2012 and 2014, (availability of data on FRS, MetS and INFLA Score) | 25 | 25 (100) | n.d. | 38.42 (12.22) | TP vs PP | n.d. | No exclusion for CVD, no data presented | Blood pressure, lipids, glucose, inflamm markers | 8 |
| Brenes 1986(7) | CS | US | Hamarville Rehabilitation Center, Pittsburgh-PA, | 66 | 56 (84.4) | *53% >1 y | 34.8 (6.8) | TP vs PP | n.d. | n.d. | Lipids, | 9 |
| Buchholz 2009 (8) | CS | Canada | Multicenter in Ontario, 2008-2010, (has cross sectional and cohort parts | 56 | 44 (78.5) | 14.55 (10.1) | 41.85 (12.22) | TP vs PP | n.d. | Reported in another study | Blood pressure, lipids, glucose, inflamm markers | 9 |
| Campbell 2004 (9) | CS | UK | British Wheelchair Racing Association, UK | 20 | 20 (100) | n.d. | 31.4 (4.5) | TP C6-7  PP-HT1-7  PP-L >T7 | n.d. | n.d. | Glucose | 7 |
| Cardus 1992 (10) | CH | US | Community-based cohort in Galveston, Texas, no date, (included SCI >9 mos) | 96 | 96 (100) | 11.8 (4.6) | 41.2 (5.0) | TP vs PP | n.d. | No CHD | Lipids | 7 |
| Davies 2007 (11) | CC | Canada | Parkwood Hospital and St. Joseph Healthcare in Ontario, Canada, | 56 | 42 (75) | 10.7 (8.3) | 40.6 (11.9) | TP vs PP | 25% | n.d. | Inflamm markers | 8 |
| de Groot (12) | CH | Netherlands | 8 Dutch rehabilitation center, ALLRISC consortium, | 115 | 84 (73) | 22.7 (5.0) | 41.4 (14.05) | TP vs PP | 53% | n.d. | Lipids, | 9 |
| Farkas 2018 (13) | CS | US | Spinal cord injury registry, Pennsylvania, US | 47 | 38 (80.1) | 14.4 (11.6) | 48.3 (9.5) | TP vs PP | 100% | Excluded CVD, DM, smokers | Blood pressure, lipids, glucose, inflammatory markers | 8 |
| Frost 2005 (14) | CS | US | Cleveland Clinic, Case Western University | 34 | 34 (100) | 10.79 (8.18) | 43.09 (11.90) | TP vs PP | n.d. | n.d. | Inflammatory markers | 8 |
| Gibson 2008 (15) | CS | Canada | Community-based cohort in Ontario, SHAPE-SCI consortium | 69 | 56 (81.1) | 14.7 (10.4) | 42.4 (12.0) | TP vs PP | 38% | n.d. | Blood pressure, lipids, glucose, inflammatory markers | 9 |
| Gorgey 2011A (16) | CS | US | Veterans Affairs Medical Center, Virginia, US | 32 | 32 (100) | n.d. | 36 (9) | TP vs PP | 100% | CVD and DM excluded | Lipids, glucose | 8 |
| Gorgey 2011B (17) | CS | US | Veterans Affairs Medical Center, Virginia, US | 13 | 13 (100) | 12.0 (7.6) | 35.2 (8.6) | TP vs PP | 100% | CVD and DM excluded | Lipids, glucose | 8 |
| Grimm 1997 (18) | CS | US | Bronx Veterans Affairs Medical Center, NY, US | 30 | 30 (100) | 10.6 (1.8) | 37 (2.1) | TP <C7  PP > T7 | 33.3% | n.d. | Blood pressure | 8 |
| Heidbreder 1982 (19) | CS | Germany | Universitatklinik Wurzburg, Frankfurt, Germany | 20 | 14 (70%) | *0.5-16y | *20-57y | C3-T1 TP  T8-L2 PP | n.d. | No CAD, HTN, DM | Blood pressure | 7 |
| Huang 2008 (20) | CS | Taiwan | Kaoshiung Medical University Hospital, Taiwan, April 2006=September 2007 | 42 | 38 (90.5) | 3.7 (2.5) | 38.1 (8.6) | TP vs PP | 52.4% | CVD and DM excluded | Lipids, glucose, inflammatory markers | 8 |
| Janssen 1997 (21) | CS | Netherlands | Vriej Universitet, Amsterdam, Netherlands | 37 | 37 (100) | 14.7 (8.6) | 37.4 (12.0) | TP C4-C8  PP-H T1-T5  PP-M T6-T10  PP-L*T11-L5 | 62% | No DM, 1 CAD, no CVD meds | Blood pressure, lipids, glucose, inflammatory markers | 8 |
| Katzelnick 2017 (22) | CS | US | Kessler foundation, NJ, and Veterans Affairs Medical Center, NY | 46 | 38 (82.6) | 10.6 (6.96) | 35.3 (7.0) | H C3-T5  L T7-12 | 45.6% | n.d. | Blood pressure | 8 |
| Katzelnick 2019 (23) | CS | US | Kessler foundation, NJ, and Veterans Affairs Medical Center, NY | 113 | 97 (85.8) | 16.9 (13.9) | 51.4 (12) | TP C1-T1  PP-H T2-4  PP-L T5-12 | 40.7% | n.d. | Blood pressure | 8 |
| Kemp 2000 (24) | CS | US | Rancho Los Amigos National Rehabilitation Center in California | 188 | 152 (81) | 16 (9) | 42 (11) | TP vs PP | 68% | n.d. | Lipids | 8 |
| Kim 2016 (25) | CS | US | Elite paracyclists participating in 2014 United Cycliste Internationale Para-Cycling Road World Cycling Championships | 44 | 30 (68) | n.d. | 39.8 (8.7) | TP C1-C8  PP T1-5  PP >T6 | n.d. | No CV disease | Blood pressure | 8 |
| King 1994 (26) | CS | US | Helen Hayes Hospital, New York, outpatient cases | 20 | 18 (90) | >1 year | 34.2 (9.8) | TP, PP-H T1-6  PP-L >T6 | n.d. | No CV disease, excluded with CV meds | Blood pressure | 8 |
| Kjaer 2001 (27) | CT | Denmark | University of Copenhagen, Denmark | 10 | 8 (80) | n.d. | *27-45y | C5-6 TP  T4-12 | n.d. | n.d. | Blood pressure | 8 |
| Kooner 1988 (28) | CT | UK | United Kingdom (hospital not specified) | 12 | 12 (100) | n.d. | n.d. | C4-6 TP  T12-L3 PP | 100% | n.d. | Blood pressure | 7 |
| Krum 1989 (29) | CC | Australia | Austin Hospital, Victoria, Australia | 20 | 17 (85) | 50% <6 mos of injury | 29.8 (3.4) | SCI-H >T6  SCI-L <T6 | ? | No meds use | Blood pressure | 8 |
| Legramante 2001 (30) | CT | Italy | CTO Hospital – Universita di Roma | 16 | n.d. | *1-19 y | 27 (7.35) | C4-7 TP  PP T/L/S | 100% | No CV disease, no CV meds | Blood pressure | 8 |
| Laclaustra 2014 (31) | CS | Spain | Miguel Servet Hospital, Aragon-ES | 177 | 133 (75.1) | 13.6 (8.1) | 40.6 (9.9) | C, T, LS | 63.9% | n.d. | Lipids | 9 |
| Matos-Souza 2010 (32) | CS | Brazil | Rehabilitation center in Sao Paulo, Brazil | 34 | 34 (100) | 6.7 (0.8) | 31.9 (1.3) | TP vs PP | 94% | No CV disease, no CV meds | Blood pressure, lipids, glucose, inflammatory markers | 8 |
| Miyatami 2014 (33) | CS | Canada | Toronto Rehabilitation Institute, Toronto, Canada | 87 | 65 (74.7) | 13.5 (4.6) | 48.1 (13.7) | TP C28  PP T1-12 | n.d. | TP (81% HTN, DL and DM) PP 16% HTN, 24% DL, 6% DM) | Blood pressure, lipids, glucose | 9 |
| O Brien 2017 (34) | CS | US | McGuire VA Medical Center | 22 | 22 (100) | 8.2 (7.9) | 36.1 (10.0) | TP vs PP | 72% | No CVD and meds | Blood pressure, lipids, glucose | 8 |
| Raymond 2010 (35) | CS | Australia | New South Wales, Australia (excluded all patients with cardiovascular morbidity) | 25 | 20 (80) | 11.0 (7.6) | 37 (9) | TP C2-8  PP-H T1-5  PP-L T6-12 | 60% | No CVD, excluded | Blood pressure, glucose | 8 |
| Sabour 2013 (36) | CS | Iran | Brain and Spinal Injury Research Center at Tehran University of Medical Sciences, Iran, May 2008 - June 2009 | 162* | 131 (80.9) | 8.03 (5.75) | 34.17 (8.75) | TP vs PP | 60% | n.d. | Blood pressure, lipids, glucose, | 8 |
| Schmid 2000 (37) | CS | Germany | University of Freiburg, Germany | 80 | 80 (100) | 12.2 (5.7) | 34.3 (8.6) | TP >T1  HPP T1-T5  LPP <T5 | 100% | n.d. | Lipids | 8 |
| Schmid 2008 (38) | CS | Germany | University of Freiburg, Germany | 112 | 80 (71.4) | 11.5 (4.4) | 33 (6.9) | TP C4-C8  PP T1 below | 100% | No CVD, excluded | Lipids | 8 |
| Sisto 2012 (39) | CS | Canada | 7 NeuroRecovery Network Rehabilitation (Boston Medical Center, Boston, MA; Frazier Rehab Institute, Louisville, KY; Kessler Institute for Rehabilitation, West Orange, NJ; Magee Rehabilitation Hospital, Philadelphia, PA; The Ohio State University Medical Center, Columbus, Ohio; Shepherd Center, Atlanta, GA; and The Institute for Rehabilitation and Research, Houston, TX.) | 350 | 267 (76.2) | 0.9 (0.1) | 42 (16) | C C1-C8  T-High T6  T Low T6 | 0% | n.d. | Blood pressure | 9 |
| Wang 2007 (40) | CS | Taiwan | University Hospital in Taiwan | 89 | 89 (62) | 10.8 (0.7) | 39.3 (1.1) | TP C8 above  PP Below T1 | 100% complete | No DM, endocrine dsease, excluded, No CV meds | Blood pressure, lipids, glucose, inflammatory markers | 8 |
| Wecht 2001 (41) | CT | US | Bronx Veterans Affairs Medical Center | 24 | 24 (100) | 13.5 (7.6) | 41 (5.5) | TP <C8  PP >T10 | 30% | No CVD, meds not checked | Blood pressure | 9 |
| Wong 2001 (42) | CS | Taiwan | Changung Memorial Hospital, Taiwan | 77 | 64 (83) | 1yr | 42.6 (14.5) | Cervical vs Thoracic vs Lumboscral | 40.3% | Excluded CV diseases | Lipid profile | 8 |
| Wecht 2006 (43) | CT | US | Bronx Veterans Affairs Medical Center | 14 | 14 (100) | 13.5 (9.8) | 38 (10.2) | TP C3-7  PP T6-12 | 50% | No CVD, excluded, no meds reported | Blood pressure | 9 |
| Yahiro 2019 (44) | CS | US | Edward Hines, Jr. Veterans Affairs (VA) Hospital in Hines, Illinois, US | 155 | 155 (100) | 18.7 (14.3) | 61.1 (13.7) | TP vs PP | 29.0% | 62% taking meds for DM, HTN and/or hyperlipidemia | Blood pressure, lipids, glucose | 8 |
| Zhong 1995 (45) | CS | US | Veterans Affairs Medical Center, NY, USA | 197 | 197 (100) | 18 (1) | 50 (1) | TP vs PP | n.d. | n.d. | Lipids | 8 |
| Zhou 1997 (46) | CT | China | China Rehabilitation Center, Beijing China | 30 | 25 (83.3) | n.d. | 28.7 (10.5) | TP <T5  PP >T5 | 100% | No HTN | Blood pressure | 7 |
| Zhu 2013 (47) | CS | US | Veterans Affairs Medical Center, NY, USA | 277 | 271 (98) | 18 (15) | 63 (14) | TP C1-8  PP-H T1-6  PP-L T7-12 | n.d. | Hypertensive meds TP 54.4%. PP-H 54.4%, PP-L 53.5% | Blood pressure | 9 |

*n.d., no data; PP, paraplegia; TP, tetraplegia; PP-H, paraplegia-high; PP-L, paraplegia-low; SCI, spinal cord injury; SCI-H, spinal cord injury high; SCI-L, spinal cord injury low; CVD, cardiovascular disease; CAD, coronary artery disease; T/L/S, thoracic, lumbar or sacral spinal levels; HTN, hypertension; DM, diabetes mellitus

Table A2. Characteristics of studies not included in the data abstraction

| **Author-Year** | **Study design (Location)** | **Participants (% Male)** | **Age, mean y (±SD )** | **SCI lesion duration (mean y±SD )** | **Outcomes** | **Results comparing intermediate cardiovascular risks** | **Reason for non-abstraction of data** |
| --- | --- | --- | --- | --- | --- | --- | --- |
| Campagnolo 2000 (48) | CC (US) | 18 (61.1) | 33.2 (8.8) | 5.6 (4.1) | Inflammatory markers | Comparison between tetraplegia and paraplegia on the immune cells. No significant difference was found between tetraplegic and paraplegic. Total white cells, granulocytes, monocytes, lymphocytes, B cell, T cell and other T cell subsets have no difference between two groups. NK cell activity was also measured with no significant difference | Measurement of inflammatory markers were unique and not present in other report |
| Groah 2011 (49) | CS (US) | 121 (80.2) | 37 (12) | 11 (8) | Blood pressure, lipids, glucose | Systolic BP was lower in tetraplegia (99.46 vs 120).  Total cholesterol is higher in paraplegia (188.82 vs 171.67 mg/dL). HDL was higher in paraplegia (45.97 mg/dL vs 42.10). LDL was higher in paraplegia (116.90 mg/dL vs 108.71).  Fasting glucose and insulin was higher in paraplegia (90.16 mg/dL vs 87.29; 10.7 microIU/mL vs 8.7)  Body mass index was higher in paraplegia (25.73 kg/m2 vs 24.46) | Report only gave the mean of the groups. No standard deviation was provided. |
| Kamakshi 2019 (50) | (CS) India | 100 (84) | 36.2 (10.9) | n.a. | Lipids, blood glucose | Dyslipidemia is found in 32.9% in tetraplegia and 67.1 in paraplegia, abnormal hbA1C (>6.5%) is found 75% in tetraplegia and 25% in paraplegia. Hyperglycemia is also more common in tetraplegia and paraplegia | No aggregated data presented for each level |
| Kanyilmaz 2013 (51) | CS (Turkey) | 34 (35.3) | 33.1 (12.1) | 1.3 (1.3) | Inflammatory markers | Phagocytosis activity (oxidative burst) and neutrophil activity of SCI patients were studied. There is decreased phagocytosis activity of neutrophils with decreased MIF (E coli) for SCI. Between tetraplegia and paraplegia, no significant difference was found | Measures were novel and not present in other report |
| Kliesch 1996 (52) | CC (US) | 49 (n.d.) | *25y | *2 wks | Inflammatory markers | NK cell mediated cell lysis mediated therapy was less for cervical therapy than thoracic injury at baseline. With rehabilitation, NK activity increased from 15.2% to 28.4% in cervical injury, and increased from 26.8% to 43.7% in thoracic group. | Values were expressed in graphs with no exact values mentioned in the report. Also, measures were new and was not present in other reports |
| Krum 1992 (53) | CC (Australia) | 327 (84) | *majority | *>10y, 29% | Blood pressure. lipids, glucose | There were no significant difference between the level of lesion, and lipids and blood pressure | Results were expressed as a graph with no estimates reported. |
| Lee 2006 (54) | CS (US) | 168 (89) | 50.2 (12.8) | 19.1 (13) | Blood pressure | Persons with paraplegia has significantly higher systolic BP, diastolic BP and mean arterial pressure. | No values were reported. Values were only shown in graphs. |
| Lee 2017 (55) | CC (South Korea) | 15 (87) | 37.78 (11.23) | 12.3 (6.0) | Blood pressure | Mean arterial pressure was 83.3 mmHg ± 21.4 for high SCI and 83.6 mmHg ± 20.3 for low SCI | Classification of exposure was above T6 (high SCI) and below T6 (low SCI) |
| Liang 2008 (56) | CS (US) | 129 (100) | 37 (4.0) | n.d. | Lipids, inflammatory marker | No differences were noted in HDL and CRP according to different levels of injury. | Classification of exposure was above T6 (high SCI) and below T6 (low SCI). Outcomes were disaggregated into different levels of injury according to CRP levels. |
| Maruyama 2008 (57) | CS (Japan) | 44 (100) | 57 (13) | 22.3 (10.5) | Blood pressure, lipids, glucose, inflammatory markers | Tetraplegia have lower systolic BP and diastolic BP compared to paraplegia. Body morphology, lipid profile and glucose have no significant different between tetraplegia and paraplegia. | Exact measurement for tetraplegic and paraplegic were not reported. |
| Ozkul 2007 (58) | CS (Turkey) | 43 (65.1) | 33.84 (10.4) | 32.56% <1y | Glucose | There were no significant difference on insulin, C peptide and oral glucose tolerance test between tetraplegia and paraplegia. | Exact measurement for tetraplegic and paraplegic were not reported. |
| Rankin 2017 (59) | CS (US) | 22 (100) | 36.1 (10.0) | 32.56% 1-5y | Blood pressure, lipids, glucose, Inflammatory markers | BMI is lower in tetraplegia than paraplegia (23.3 kg/m2 ± 4.5 vs 25.3 ± 3.4).  Total cholesterol, triglycerides and LDL is higher in tetraplegia than in paraplegia (160.4 mg/dL ± 21.1 vs 144.3 ± 31.9; 141.1 ± 68.2 vs 93.9 ± 37.6; 98.4 ± 23.2 vs 89.3 ± 29.1)  Fasting glucose and HbA1C is higher in tetraplegia than paraplegia (114.5 mg/dL ± 14.54 vs 102.5 ± 9.8; 5.61% ± 0.50 vs 5.19 ± 0.34)  TNF alpha is higher in tetraplegic versus paraplegic (16.0 pg/mL ± 8.2 vs 14.9 ± 8.1) | Study characteristics are similar with O’Brien, et. al. 2017. This was assumed to be of similar study reporting similar outcomes. |
| Storch 2005 (60) | CS (Germany) | 32 (0) | 29.9 (6.3) | 34.88% >5y | Lipids | No significant difference was observed in the lipoproteins between tetraplegia and paraplegia | Study characteristics are similar with Schmid, et. al., 2008. This was assumed to be of similar study reporting a subset of an outcome (only females) |
| Vidal 2003 (61) | CS (Spain) | 2135 (72) | 50.08 (16.25) | 8.2 (7.9) | Lipids, glucose | Total cholesterol HDL and LDL are significantly different from one another, with higher levels for lower levels of injury. | The exposure has different classification (i.e., group 1 from cervical to T5, group 2 from T6 to T12, and group 3 from lumbar and below) |
| Wang 2009 (62) | CS (Taiwan) | 69 (100%) | 40 (11) | 10.6 (7.1) | Lipids, inflammatory markers | Post-challenge hyperglycemia and serum albumin levels are two important indicators of cardiovascular health in men. | Study characteristics are similar with Wang, et. al. 2009. This was assumed to be of similar study reporting similar outcomes. |
| Vaziri 1982 (63) | CS (US) | 10 (100) | 59 (8.8) | n.d. | Lipids | Serum triglycerides were elevated for chronic renal failure group | Outcomes were reported per individual |
| Yamamotova 2010 (64) | CS (Czech Republic) | 42 (83.3) | 38.1 (17.4) | 12 (7) | Lipids, glucose, inflammatory markers | Total cholesterol and triglycerides are higher in paraplegic compared to tetraplegic (3.98 ± 0.79 vs 3.31 ± 0.88; 1.26 ± 0.59 vs 1.01 ± 0.61)  Glucose are higher in paraplegia compared to tetraplegia (8.72 ± 2.10 vs 7.69 ± 2.22) | The study focused on acute SCI, with all participants with less 2 months from the injury. |
| Yoo 1997 (65) | CT (South Korean) | 106 (88.6) | 46 (17) acute 40 (14) chronic TP | 26.8 (7.4) | Blood pressure | Systolic arterial pressure are lower in acute paraplegia compared to acute tetraplegia | No values were reported. Values were only shown in graphs. |

*n.d., no data; PP, paraplegia; TP, tetraplegia, HDL, high density lipoprotein; LDL, low-density lipoprotein

Table A3-a. Subgroup analysis by study characteristics (tetraplegia vs paraplegia)

| Study characteristics | Stratum | Number of studies | Weighted mean difference | I2 for heterogeneity | Heterogeneity by metaregression |
| --- | --- | --- | --- | --- | --- |
| Systolic Blood Pressure (mmHg) | | | | | |
| *Individual factors* | | | | | |
| Sex | All male | 7 | -18.377 (-28.260, -8.495) | 95.6% | 0.243 |
|  | Mixed | 12 | -12.404 (-16.838, -7.970) | 94.8% |  |
| Body mass index | ≤ Median | 9 | -11.407 (-18.683, -4.130) | 94.3% | 0.722 |
|  | > Median | 7 | -12.969 (-19.378, -6.560) | 86.4% |  |
| Age | ≤ Median | 10 | -13.299 (-21.289, -5.308) | 95.4% | 0.973 |
|  | > Median | 9 | -12.939 (-18.156, -7.722) | 84.2% |  |
| Baseline CV risk/meds | No CV disease | 11 | -17.155 (-24.094, -10.216) | 91.1% | 0.325 |
|  | With CV disease | 6 | -11.701 (-17.542, -5.861) | 83.2% |  |
| *Injury factors* | | | | | |
| Duration of injury | ≤ Median | 8 | -12.017 (-20.661, -3.374) | 95.5% | 0.846 |
|  | > Median | 7 | -12.328 (-17.244, -7.413) | 79.5% |  |
| Completeness of lesion | Mixed | 11 | -10.785 (-16.241, -5.330) | 88.7% | 0.486 |
|  | 100% complete | 3 | -16.993 (-33.752, -0.235) | 93.3% |  |
| *Study design factors* | | | | | |
| Position | Supine | 4 | -12.350 (-28.449, 3.749) | 93.0% | 0.918 |
|  | Seated | 11 | -13.155 (-18.842, -7.467) | 88.3% |  |
| Location | Asia | 4 | -5.573 (-12.647, 1.501) | 80.8% | 0.077 |
|  | Europe | 4 | -23.342 (-30.072, -16.612) | 81.1% |  |
|  | North America | 11 | -11.238 (-15.641, -6.836) | 79.2% |  |
|  | South America | 1 | -22.100 (-24.880, -19.320) | - |  |
| Participant | ≤ Median | 10 | -19.964 (-26.778, -13.150) | 91.5% | **0.011***** |
|  | > Median | 10 | -7.883 (-11.936, -3.829) | 83.2% |  |
| Quality | High (≥ 8) | 18 | -13.646 (-18.506, -8.785) | 93.1% | 0.890 |
|  | Moderate (< 8) | 2 | -14.876 (-29.557, -0.194) | 90.5% |  |
| Diastolic Blood Pressure (mmHg) | | | | | |
| *Individual factors* | | | | | |
| Sex | All male | 7 | -8.524 (-12.830, -4.218) | 86.8% | 0.480 |
|  | Mixed | 12 | -6.728 (-9.372, -4.085) | 78.8% |  |
| Body mass index | ≤ Median | 9 | -6.544 (-10.681, -2.406) | 89.4% | 0.929 |
|  | > Median | 7 | -6.675 (-9.768, -3.581) | 70.1% |  |
| Age | ≤ Median | 9 | -6.704 (-10.795, -2.613) | 88.4% | 0.804 |
|  | > Median | 11 | -7.282 (-10.267, -4.297) | 74.6% |  |
| Baseline CV risk/meds | No CV disease | 11 | -8.095 (-10.964, -5.225) | 65.5% | 0.429 |
|  | With CV disease | 5 | -9.334 (-12.568, -6.099) | 58.9% |  |
| *Injury factors* | | | | | |
| Duration of injury | ≤ Median | 8 | -6.190 (-10.707, -1.673) | 89.3% | 0.569 |
|  | > Median | 7 | -7.777 (-11.164, -4.391) | 79.5% |  |
| Completeness of lesion | Mixed | 11 | -5.624 (-8.612, -2.637) | 79.8% | 0.490 |
|  | 100% complete | 3 | -8.033 (-14.868, -1.198) | 68.7% |  |
| *Study design factors* | | | | | |
| Position | Supine | 4 | -6.620 (-12.865, -0.374) | 93.0% | 0.918 |
|  | Seated | 11 | -6.424 (-9.583, -3.264) | 88.3% |  |
| Location | Asia | 4 | -2.720 (-7.277, 1.837) | 54.7% | **0.007***** |
|  | Europe | 4 | -10.463 (-14.611, -6.315) | 75.0% |  |
|  | North America | 11 | -6.075 (-8.174, -3.976) | 53.5% |  |
|  | South America | 1 | -12.000 (-13.950, -10.050) | - |  |
| Participant | ≤ Median | 11 | -6.170 (-9.977, -2.362) | 70.2% | 0.135 |
|  | > Median | 9 | -5.516 (-8.684, -2.348) | 84.4% |  |
| Quality | High (≥ 8) | 18 | -7.217 (-9.701, -4.734) | 83.6% | 0.635 |
|  | Moderate (< 8) | 2 | -6.504 (-9.088, -3.921) | 8.5% |  |
| Triglycerides (mg/dL) | | | | | |
| *Individual factors* | | | | | |
| Sex | All male | 10 | -14.377 (-26.373, -2.382) | 97.0% | 0.357 |
|  | Mixed | 13 | -6.831 (-16.187, 2.524) | 0.0% |  |
| Body mass index | ≤ Median | 9 | -2.769 (-16.654, 11.117) | 62.1% | 0.440 |
|  | > Median | 11 | -11.004 (-26.562, 4.553) | 93.3% |  |
| Age | ≤ Median | 8 | -3.771 (-22.555, 15.013) | 87.8% | 0.397 |
|  | > Median | 12 | -14.791 (-26.454, -3.129) | 95.6% |  |
| Baseline CV risk/meds | No CV disease | 9 | -4.514 (-26.287, 17.258) | 89.5% | **0.004***** |
|  | With CV disease | 3 | -12.337 (-31.958, 7.283) | 0.0% |  |
| *Injury factors* | | | | | |
| Duration of injury | ≤ Median | 8 | -7.075 (-30.079, 15.930) | 90.7% | 0.865 |
|  | > Median | 11 | -11.294 (-26.315, 3.726) | 93.4% |  |
| Completeness of lesion | Mixed | 5 | -15.862 (-28.350, -3.373) | 0.0% | 0.124 |
|  | 100% complete | 10 | 0.045 (-12.658, 12.749) | 37.3% |  |
| *Study design factors* | | | | | |
| Location | Asia | 4 | -17.834 (-29.582, -6.086) | 0.0% | 0.279 |
|  | Europe | 4 | 4.379 (-21.903, 30.662) | 55.9% |  |
|  | North America | 13 | -14.654 (-25.911, -3.396) | 95.6% |  |
|  | South America | 1 | 10.300 (0.326, 20.274) | - |  |
| Participant | ≤ Median | 11 | 5.546 (-4.191, 15.283) | 13.6% | **0.004***** |
|  | > Median | 10 | -20.765 (-31.729, -9.802) | 96.2% |  |
| Quality | High (≥ 8) | 18 | -6.319 (-12.401, -0.237) | 70.5% | **<0.000***** |
|  | Moderate (< 8) | 2 | -46.986 (-51.221, -42.752) | 0.0% |  |
| Total cholesterol (mg/dL) | | | | | |
| *Individual factors* | | | | | |
| Sex | All male | 9 | -9.010 (-15.315, -2.705) | 96.2% | 0.646 |
|  | Mixed | 11 | -11.188 (-18.174, -4.201) | 48.9% |  |
| Body mass index | ≤ Median | 12 | -6.474 (-10.082, -2.866) | 56.4% | 0.367 |
|  | > Median | 6 | -13.221 (-22.767, -3.676) | 52.0% |  |
| Age | ≤ Median | 8 | -10.992 (-21.021, -0.963) | 50.6% | 0.709 |
|  | > Median | 13 | -9.394 (-14.668, -4.120) | 94.5% |  |
| Baseline CV risk/meds | No CV disease | 8 | -8.228 (-16.018, -0.438) | 41.5% | 0.834 |
|  | With CV disease | 3 | -6.511 (-22.770, 9.749) | 37.5% |  |
| *Injury factors* | | | | | |
| Duration of injury | ≤ Median | 7 | -9.756 (-18.636, -0.876) | 62.2% | 0.912 |
|  | > Median | 11 | -7.928 (-11.598, -4.258) | 64.0% |  |
| Completeness of lesion | Mixed | 5 | -13.697 (-19.635, -7.760) | 0.0% | 0.692 |
|  | 100% complete | 10 | -10.437 (-19.742, -1.131) | 58.7% |  |
| *Study design factors* | | | | | |
| Location | Asia | 4 | -15.065 (-23.267, -6.863) | 10.2% | 0.562 |
|  | Europe | 4 | -11.748 (-22.006, -1.489) | 28.8% |  |
|  | North America | 13 | -8.239 (-13.830, -2.648) | 94.6% |  |
|  | South America | - | - |  |  |
| Participant | ≤ Median | 8 | -5.812 (-13.659, 2.035) | 12.4% | 0.310 |
|  | > Median | 13 | -11.263 (-16.529, -5.997) | 94.7% |  |
| Quality | High (≥ 8) | 19 | -11.142 (-16.504, -5.780) | 90.7% | 0.228 |
|  | Moderate (< 8) | 2 | -3.159 (-5.113, -1.206) | 0.0% |  |
| High density lipoprotein (mg/dL) | | | | | |
| *Individual factors* |  |  |  |  |  |
| Sex | All male | 10 | -1.770 (-3.867, 0.328) | 98.1% | 0.852 |
|  | Mixed | 11 | -1.684 (-3.518, 0.149) | 30.2% |  |
| Body mass index | ≤ Median | 8 | -3.079 (-4.697, -1.461) | 33.4% | **0.011***** |
|  | > Median | 11 | 0.002 (-1.524, 1.528) | 89.9% |  |
| Age | ≤ Median | 11 | -2.790 (-4.224, -1.356) | 46.6% | 0.286 |
|  | > Median | 11 | -0.968 (-3.082, 1.146) | 97.5% |  |
| Baseline CV risk/meds | No CV disease | 9 | -2.933 (-4.238, -1.627) | 27.6% | **0.033***** |
|  | With CV disease | 3 | 0.838 (-2.392, 4.069) | 0.0% |  |
| *Injury factors* |  |  |  |  |  |
| Duration of injury | ≤ Median | 8 | -2.753 (-4.143, -1.363) | 42.0% | **0.016***** |
|  | > Median | 5 | -0.001 (-1.513, 1.510) | 90.0% |  |
| Completeness of lesion | Mixed | 5 | -1.181 (-2.909, 0.547) | 0.0% | 0.353 |
|  | 100% complete | 10 | -2.334 (-4.372, -0.296) | 33.5% |  |
| *Study design factors* |  |  |  |  |  |
| Location | Asia | 4 | -1.804 (-3.518, -0.090) | 0.0% | 0.648 |
|  | Europe | 4 | -0.616 (-5.358, 4.126) | 58.9% |  |
|  | North America | 13 | -1.541 (-3.487, 0.405) | 97.2% |  |
|  | South America | 1 | -5.300 (-7.036, -3.564) | - |  |
| Participant | ≤ Median | 12 | -3.805 (-5.004, -2.607) | 0.0% | 0.244 |
|  | > Median | 10 | -0.947 (-2.984, 1.090) | 97.9% |  |
| Quality | High (≥ 8) | 20 | -2.013 (-3.970, -0.055) | 93.9% | 0.383 |
|  | Moderate (< 8) | 2 | 0.460, (-4.587, 5.506) | 98.3% |  |
| Low density lipoprotein (mg/dL) | | | | | |
| *Individual factors* | | | | | |
| Sex | All male | 10 | -3.386 (-7.222, 0.451) | 88.7% | 0.056 |
|  | Mixed | 10 | -9.716 (-15.199, -4.233) | 37.7% |  |
| Body mass index | ≤ Median | 10 | -6.359 (-11.260, -1.459) | 85.5% | 0.383 |
|  | > Median | 9 | -10.568 (-16.863, -4.273) | 18.8% |  |
| Age | ≤ Median | 10 | -8.458 (-17.447, 0.531) | 82.8% | 0.332 |
|  | > Median | 10 | -4.016 (-7.553, -0.478) | 84.1% |  |
| Baseline CV risk/meds | No CV disease | 9 | -6.272 (-13.274, 0.730) | 83.3% | 0.958 |
|  | With CV disease | 3 | -5.906 (-17.999, 6.187) | 45.3% |  |
| *Injury factors* | | | | | |
| Duration of injury | ≤ Median | 10 | -8.520 (-17.056, 0.016) | 83.5% | 0.597 |
|  | > Median | 8 | -4.751 (-9.350, -0.151) | 83.2% |  |
| Completeness of lesion | Mixed | 9 | -7.754 (-12.640, -2.868) | 32.1% | 0.105 |
|  | 100% complete | 5 | -16.450 (-27.767, -5.133) | 36.7% |  |
| *Study design factors* | | | | | |
| Location | Asia | 3 | -9.625 (-18.079, -1.172) | 0.0% | **0.021***** |
|  | Europe | 4 | -13.299 (-24.227, -2.370) | 51.4% |  |
|  | North America | 12 | -2.273 (-5.835, 1.289) | 84.3% |  |
|  | South America | 1 | -15.300 (-21.615, -8.985) | - |  |
| Participant | ≤ Median | 10 | -8.712 (-16.726, -0.697) | 55.7% | 0.158 |
|  | > Median | 10 | -4.131 (-7.630, -0.633) | 88.0% |  |
| Quality | High (≥ 8) | 18 | -8.300 -11.639 -4.960 | 71.2% | **<0.001***** |
|  | Moderate (< 8) | 2 | 4.377 (2.448, 6.306) | 0.0% |  |
| Cholesterol-HDL ratio | | | | | |
| *Individual factors* | | | | | |
| Sex | All male | 5 | -0.126 (-0.629, 0.376) | 78.1% | 0.703 |
|  | Mixed | 4 | -0.294 (-0.634, 0.047) | 0.0% |  |
| Body mass index | ≤ Median | 4 | -0.295 (-0.607, 0.017) | 0.0% | 0.794 |
|  | > Median | 5 | -0.144 (-0.700, 0.412) | 79.1% |  |
| Age | ≤ Median | 5 | -0.056 (-0.493, 0.382) | 52.8% | 0.270 |
|  | > Median | 4 | -0.470 (-0.760, -0.181) | 27.8% |  |
| Baseline CV risk/meds | No CV disease | 5 | -0.087 (-0.561, 0.387) | 85.2% | - |
|  | With CV disease | 0 | - | 0.0% |  |
| *Injury factors* | | | | | |
| Duration of injury | ≤ Median | 4 | -0.056 (-0.566, 0.454) | 27.8% | 0.323 |
|  | > Median | 4 | -0.470 (-0.760, -0.181) | 64.4% |  |
| Completeness of lesion | Mixed | 3 | 0.163 (-0.525, 0.851) | 66.0% | 0.124 |
|  | 100% complete | 5 | -0.314 (-0.613, -0.015) | 0.0% |  |
| *Study design factors* | | | | | |
| Location | Asia | 1 | -0.300 (-0.783, 0.183) | - | 0.355 |
|  | Europe | 3 | -0.361 (-0.734, 0.013) | 0.0% |  |
|  | North America | 5 | -0.061 (-0.685, 0.563) | 80.0% |  |
|  | South America | - | - | - |  |
| Participant | ≤ Median | 4 | 0.185 (-0.357, 0.727) | 38.8% | **0.020***** |
|  | > Median | 5 | -0.573 (-0.668, -0.478) | 0.0% |  |
| Quality | High (≥ 8) | 8 | -0.125 (-0.423, 0.173) | 30.7% | 0.159 |
|  | Moderate (< 8) | 1 | -0.600 (-0.700, -0.500) | - |  |
| Fasting glucose (mg/dL) | | | | | |
| *Individual factors* | | | | | |
| Sex | All male | 6 | -0.288 (-2.170, 1.594) | 61.5% | 0.783 |
|  | Mixed | 4 | 0.169 (-3.697, 4.035) | 0.0% |  |
| Body mass index | ≤ Median | 6 | -0.084 (-1.724, 1.556) | 54.3% | 0.796 |
|  | > Median | 6 | -0.538 (-3.579, 2.503) | 0.0% |  |
| Age | ≤ Median | 7 | -0.962 (-3.086, 1.161) | 56.6% | 0.325 |
|  | > Median | 6 | 0.594 (-1.218, 2.405) | 0.0% |  |
| Baseline CV risk/meds | No CV disease | 6 | 0.748 (-1.939, 3.434) | 45.2% | 0.898 |
|  | With CV disease | 3 | 0.191 (-4.156, 4.538) | 0.0% |  |
| *Injury factors* | | | | | |
| Duration of injury | ≤ Median | 6 | 0.490 (-1.507, 2.487) | 29.3% | 0.485 |
|  | > Median | 5 | -1.025 (-2.024, -0.026) | 0.0% |  |
| Completeness of lesion | Mixed | 6 | 0.307 (-3.160, 3.773) | 23.4% | 0.782 |
|  | 100% complete | 4 | 0.305 (-5.040, 5.650) | 23.3% |  |
| *Study design factors* | | | | | |
| Location | Asia | 4 | -1.684 (-4.326, 0.958) | 0.0% | 0.804 |
|  | Europe | - | - | - |  |
|  | North America | 8 | 0.277 (-2.424, 2.979) | 23.2% |  |
|  | South America | 1 | 1.600 (-0.899, 4.099) | - |  |
| Participant | ≤ Median | 6 | 0.440 (-3.616, 4.496) | 68.3% | 0.986 |
|  | > Median | 7 | -0.802 (-1.683, 0.079) | 0.0% |  |
| Quality | High (≥ 8) | 12 | -0.002 (-1.391, 1.387) | 24.5% | 0.077 |
|  | Moderate (< 8) | 1 | -3.600 (-6.291, -0.909) | - |  |

| Insulin (microU/mL) | | | | | |
| --- | --- | --- | --- | --- | --- |
| *Individual factors* | | | | | |
| Sex | All male | 3 | -0.357 (-2.349, 1.634) | 89.7% | 0.239 |
|  | Mixed | 2 | 2.683 (-0.566, 5.933) | 0.0% |  |
| Body mass index | ≤ Median | 3 | -0.357 (-2.349, 1.634) | 89.1% | 0.239 |
|  | > Median | 2 | 2.683 (-0.566, 5.933) | 17.7% |  |
| Age | ≤ Median | 3 | -0.357 (-2.349, 1.634) | 89.7% | 0.239 |
|  | > Median | 2 | 2.683 (-0.566, 5.933) | 0.0% |  |
| Baseline CV risk | No CV disease | 2 | -1.537 (-2.410, -0.663) | 0.0% | 0.136 |
|  | With CV disease | 1 | 2.270 (-2.663, 7.203) | - |  |
| *Injury factors* | | | | | |
| Duration of injury | ≤ Median | 2 | -0.419 (-2.673, 1.834) | 94.8% | 0.294 |
|  | > Median | 2 | 2.683 (-0.566, 5.933) | 0.0% |  |
| Completeness of lesion | Mixed | 1 | 3.000 (-1.318, 7.318) | - | 0.293 |
|  | 100% complete | 2 | -1.537 (-2.410, -0.663) | 0.0% |  |
| *Study design factors* | | | | | |
| Location | Asia | 1 | -1.600 (-2.492, -0.708) | - | 0.813 |
|  | Europe | - | - | - |  |
|  | North America | 4 | 0.737 (0.241, 1.234) | 0.0% |  |
|  | South America | - | - | - |  |
| Participant | ≤ Median | 4 | 0.249 (-2.244, 2.742) | 54.4% | 0.843 |
|  | > Median | 1 | 0.700 (0.194, 1.206) | - |  |
| Quality | High (≥ 8) | 5 | 0.262 (-1.475, 1.998) | 80.1% | - |
|  | Moderate (< 8) | - | - | - |  |

***Statistically significant, p value < 0.05

Table A3-b. Subgroup analysis by study characteristics (high paraplegia vs low paraplegia)

| Study characteristics | Stratum | Number of studies | Weighted mean difference | I2 for heterogeneity | Chi square for heterogeneity |
| --- | --- | --- | --- | --- | --- |
| Systolic Blood Pressure (mmHg) | | | | | |
| *Individual factors* | | | | | |
| Sex | All male | 1 | -13.600 (-27.874, 0.674) | - | 0.621 |
|  | Mixed | 9 | -9.710 (-10.957, -8.463) | 54.3% |  |
| Body mass index | ≤ Median | 4 | -7.412 (-14.129, -0.694) | 45.2% | 0.811 |
|  | > Median | 3 | -8.745 (-14.650, -2.840) | 54.6% |  |
| Age | ≤ Median | 5 | -13.890 (-18.729, -9.052) | 0.0% | 0.116 |
|  | > Median | 5 | -7.996 (-11.120, -4.871) | 64.7% |  |
| Baseline CV risk./meds | No CV disease | 4 | -13.367 (-18.607, -8.127) | 0.0% | 0.164 |
|  | With CV disease | 3 | -7.217 (-14.167, -0.267) | 71.6% |  |
| *Injury factors* | | | | | |
| Duration of injury | ≤ Median | 4 | -10.623 (-12.780, -8.466) | 6.0% | 0.150 |
|  | > Median | 4 | -5.707 (-12.175, 0.762) | 69.5% |  |
| Completeness of lesion | Mixed | 2 | -2.729 (-11.582, 6.123) | 5.2% | 0.541 |
|  | 100% complete | 3 | -8.477 (-18.076, 1.121) | 70.4% |  |
| *Study design factors* | | | | | |
| Location | Asia | 2 | -14.254 (-22.069, -6.439) | 11.8% | 0.117 |
|  | Europe | 2 | -5.595 (-17.482, 6.292) | 60.3% |  |
|  | North America | 7 | -9.931 (-11.224, -8.637) | 0.0% |  |
|  | South America | - | - | - |  |
| Participant | ≤ Median | 6 | -13.448 (-17.682, -9.213) | 0.0% | 0.092 |
|  | > Median | 4 | -7.417 (-10.895, -3.940) | 72.7% |  |
| Quality | High (≥ 8) | 10 | -9.740 (-10.982, -8.497) | 42.2% | - |
|  | Moderate (< 8) | - | - | - |  |
| Diastolic blood pressure (mmHg) | | | | | |
| *Individual factors* | | | | | |
| Sex | All male | 1 | -6.570 (-17.012, 3.872) | - | 0.826 |
|  | Mixed | 9 | -5.369 (-7.656, -3.082) | 59.6% |  |
| Body mass index | ≤ Median | 4 | -4.010 (-7.833, -0.187) | 0.0% | 0.634 |
|  | > Median | 3 | -4.643 (-9.665, 0.379) | 83.6% |  |
| Age | ≤ Median | 5 | -4.224 (-8.311, -0.137) | 0.0% | 0.593 |
|  | > Median | 5 | -5.815 (-8.417, -3.212) | 72.6% |  |
| Baseline CV risk/meds | No CV disease | 4 | -3.440 (-8.450, 1.570) | 12.8% | 0.384 |
|  | With CV disease | 3 | -6.377 (-11.185, -1.568) | 55.0% |  |
| *Injury factors* | | | | | |
| Duration of injury | ≤ Median | 4 | -5.221 (-8.945, -1.497) | 42.4% | 0.973 |
|  | > Median | 4 | -4.643 (-9.665, 0.379) | 78.8% |  |
| Completeness of lesion | Mixed | 2 | -1.082 (-4.496, 2.333) | 0.0% | 0.243 |
|  | 100% complete | 3 | -5.117 (-9.426, -0.808) | 72.0% |  |
| *Study design factors* | | | | | |
| Location | Asia | 2 | 0.164 (-6.043, 6.372) | 0.0% | 0.244 |
|  | Europe | 2 | -3.141 (-8.358, 2.076) | 0.0% |  |
|  | North America | 6 | -6.548 (-8.866, -4.229) | 60.2% |  |
|  | South America | - | - |  |  |
| Participant | ≤ Median | 6 | -5.227 (-8.860, -1.594) | 0.0% | 0.934 |
|  | > Median | 4 | -5.477 (-8.311, -2.642) | 79.0% |  |
| Quality | High (≥ 8) | 10 | -7.060 (-9.712, -4.408) | 67.3% | 0.556 |
|  | Moderate (< 8) | - | - | - |  |

Table A4. Metaregression of intermediate cardiovascular outcome with age, body mass index and lesion duration

|  | Sex (% males) | Age | Body mass index | Complete injury % | Lesion duration |
| --- | --- | --- | --- | --- | --- |
|  | Beta (95% CI) | Beta (95% CI) | Beta (95% CI) | Beta (95% CI) | Beta (95% CI) |
| Tetraplegia vs Paraplegia | | | | | |
| Systolic blood pressure | -0.14 (-0.55, 0.27) | 0.26 (-0.26, 0.79) | 0.86 (-2.35, 4.07) | -0.11 (-0.36, 0.13) | -0.36 (-1.64, 0.94) |
| Diastolic blood pressure | 0.17 (-1.28, 1.63) | -0.03 (-0.20, 0.26) | -0.25 (-1.95, 1.45) | -0.07 (-0.17, 0.03) | -0.29 (-0.85, 0.27) |
| Triglycerides | -0.51 (-1.59, 0.57) | -0.48 (-1.82, 0.87) | -2.49 (-9.85, 4.85) | 0.19 (-0.19, 0.58) | -0.74 (-3.42, 1.93) |
| Cholesterol | 0.09 (-0.44, 0.62) | 0.14 (-0.57, 0.87) | 1.02 (-2.77, 4.83) | -0.06 (-0.34, 0.21) | -0.03 (-1.21, 1.15) |
| High density lipoprotein | -0.03 (-0.19, 0.13) | **0.18 (0.01, 0.35)*** | 0.62 (-0.48, 1.73) | -0.02 (-0.08, 0.04) | **0.37 (0.08, 0.66)*** |
| Low density lipoprotein | 0.32 (-0.13, 0.77) | 0.09 (-0.51, 0.69) | 1.51 (-1.82, 4.84) | -0.05 (-0.30, 0.19) | 0.02 (-1.20, 1.23) |
| Cholesterol:HDL | 0.01 (-0.03, 0.04) | -0.03 (-0.08, 0.03) | 0.01 (-0.22, 0.24) | -0.01 (-0.02, 0.01) | -0.05 (-0.14, 0.03) |
| Glucose | -0.02 (-0.27, 0.23) | 0.13 (-0.15, 0.42) | -0.03 (-1.17, 1.09) | 0.02 (-0.05, 0.09) | 0.01 (-0.51, 0.54) |
| Insulin | -0.14 (-0.48, 0.18) | 0.28 (-0.92, 1.48) | **0.94 (0.29, 1.60)*** | -0.07 (-0.53, 0.39) | **1.02 (0.07, 1.98)*** |
| High-Para vs. Low-Para (T6 as the level of discrimination) | | | | | |
| Systolic blood pressure | -0.12 (-0.53, 0.29) | 0.22 (-0.17. 0.61) | 0.71 (-2.26, 3.68) | 0.08 (-0.72, 0.87) | 0.39 (-0.17, 0.93) |
| Diastolic blood pressure | -0.09 (-0.41, 0.23) | -0.09 (-0.40, 0.21) | 0.27 (-1.99, 2.54) | -0.04 (-0.44, 0.35) | 0.01 (-0.49, 0.52) |

Figure A1. Forest plots of each outcome

| 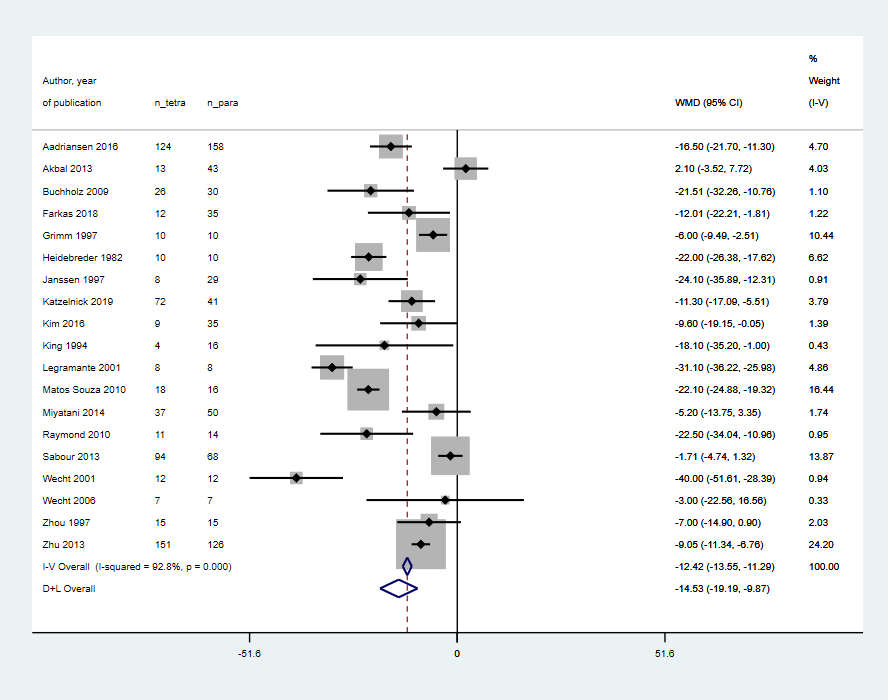  Systolic blood pressure (D-L der Simonian Laird, random-effects model) | 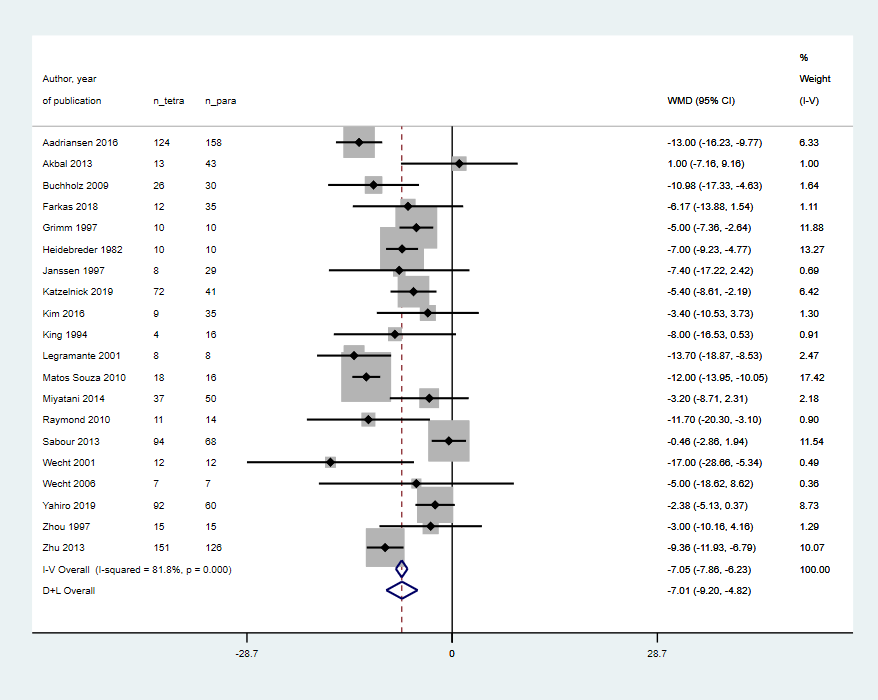  Diastolic blood pressure (D-L der Simonian Laird, random-effects model) |
| --- | --- |
| 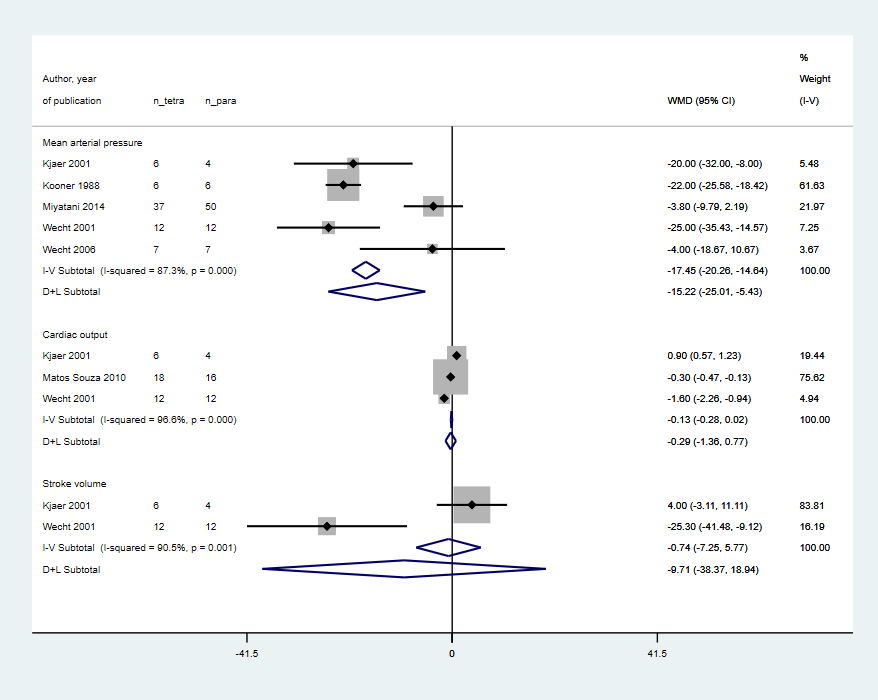  Mean arterial pressure and others (D-L der Simonian Laird, random-effects model) | 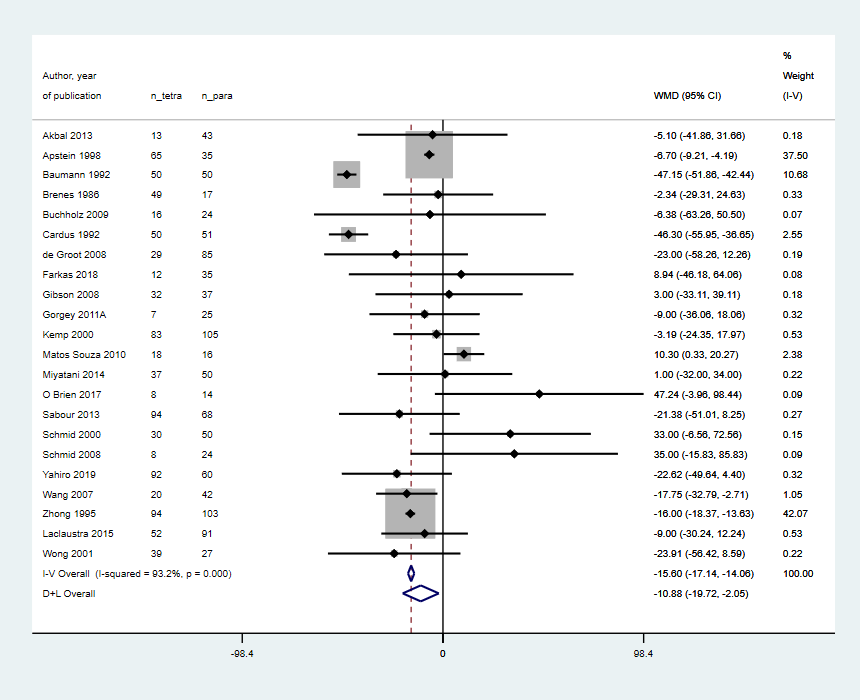  Triglycerides (D-L der Simonian Laird, random-effects model) |
| 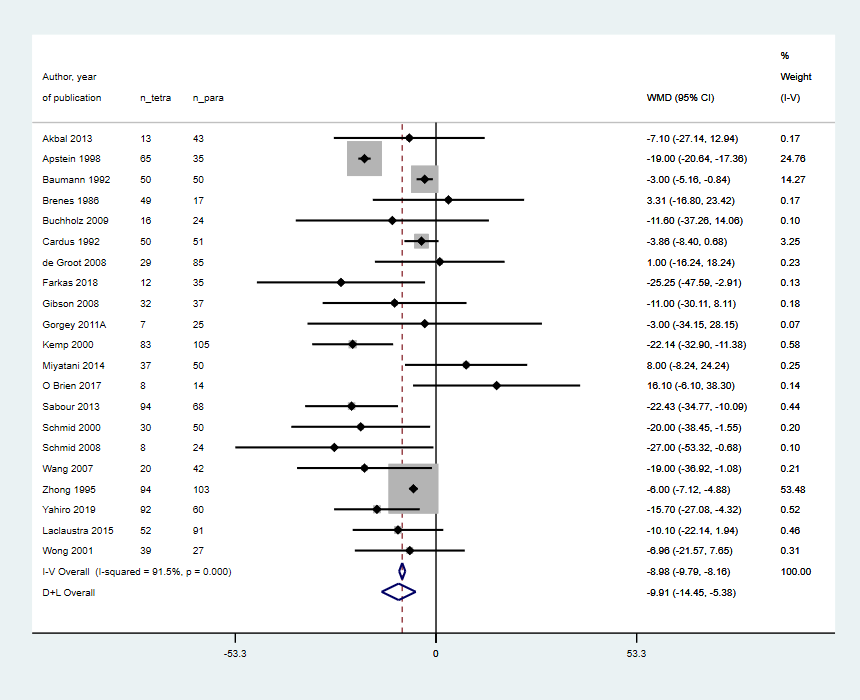  Total cholesterol (D-L der Simonian Laird, random-effects model) | 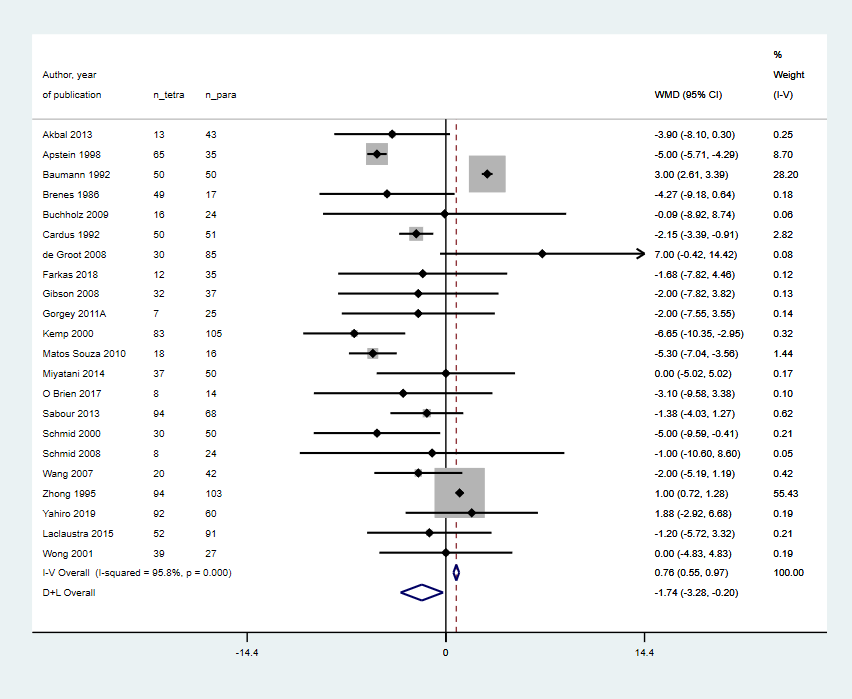  High density lipoprotein (D-L der Simonian Laird, random-effects model) |
| 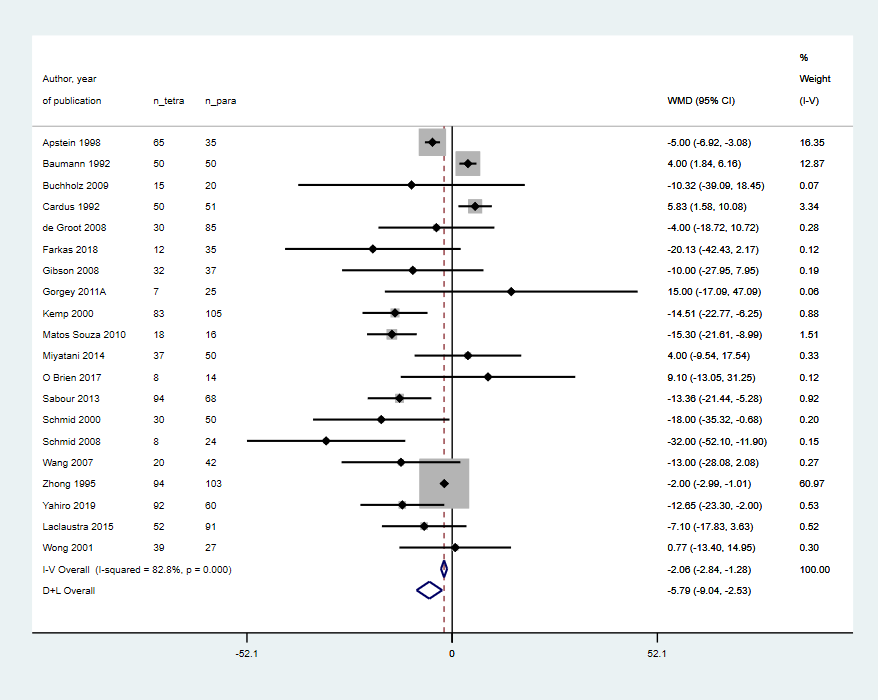  Low density lipoprotein (D-L der Simonian Laird, random-effects model) | 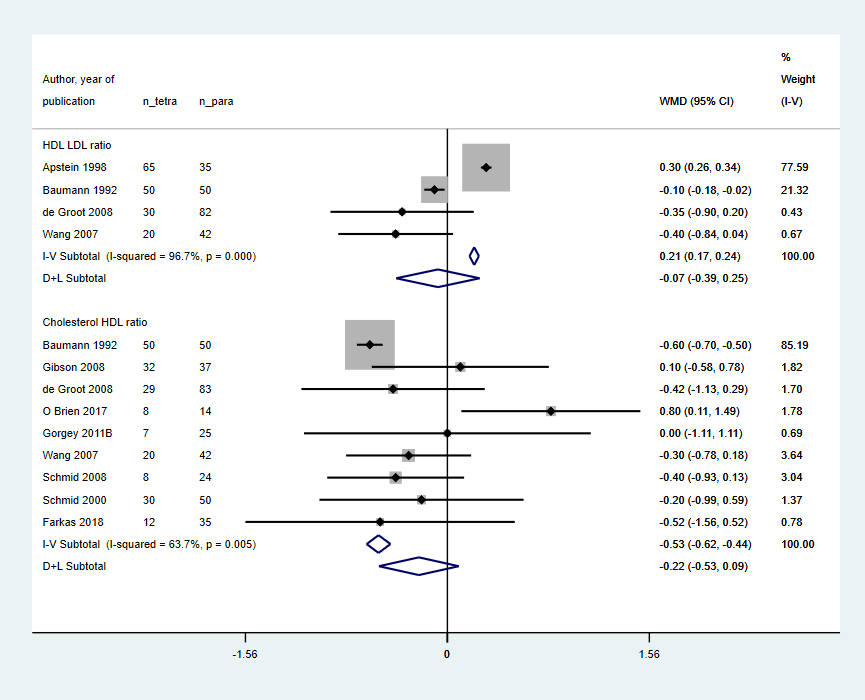  Lipid ratios (D-L der Simonian Laird, random-effects model) |
| 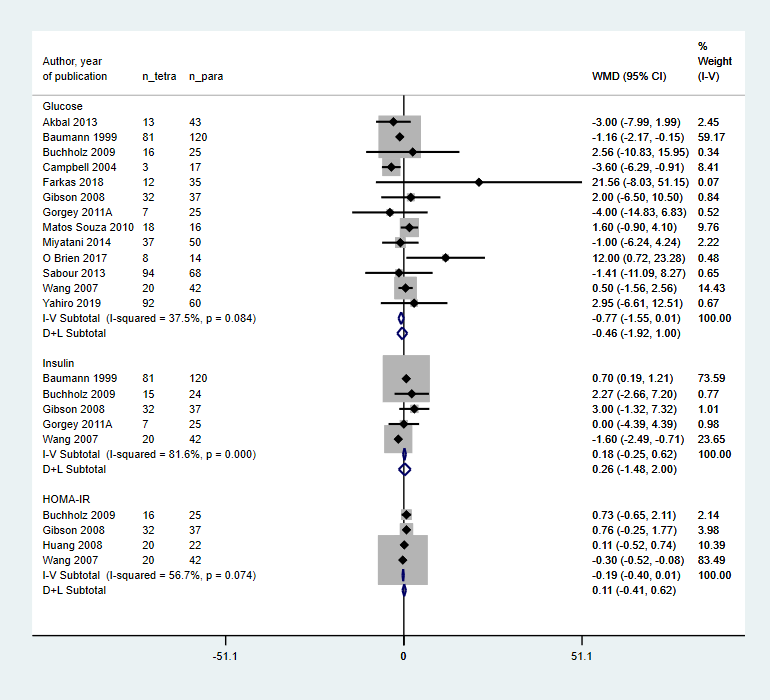  Glucose, Insulin and HOMA IR (D-L der Simonian Laird, random-effects model) | 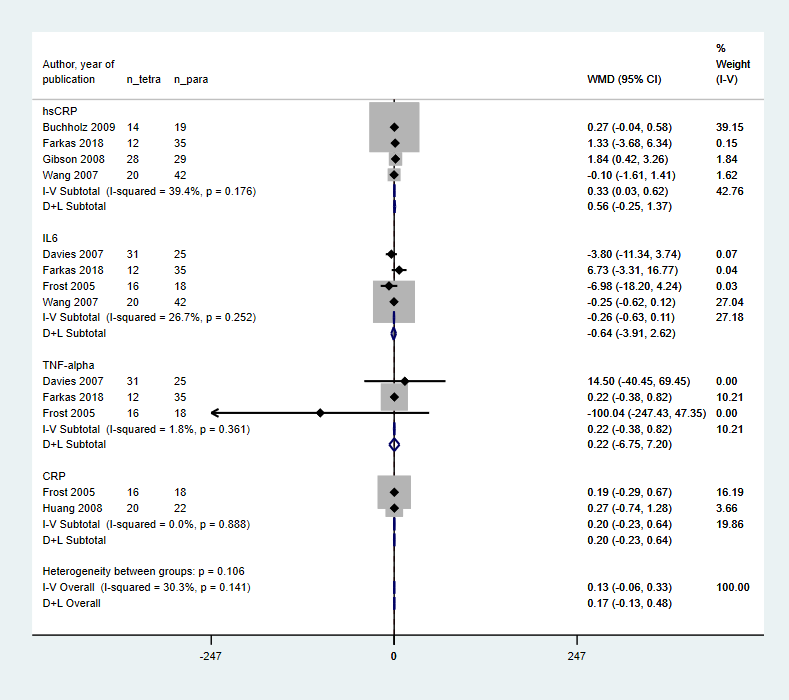  Inflammatory markers (D-L der Simonian Laird, random-effects model) |
| 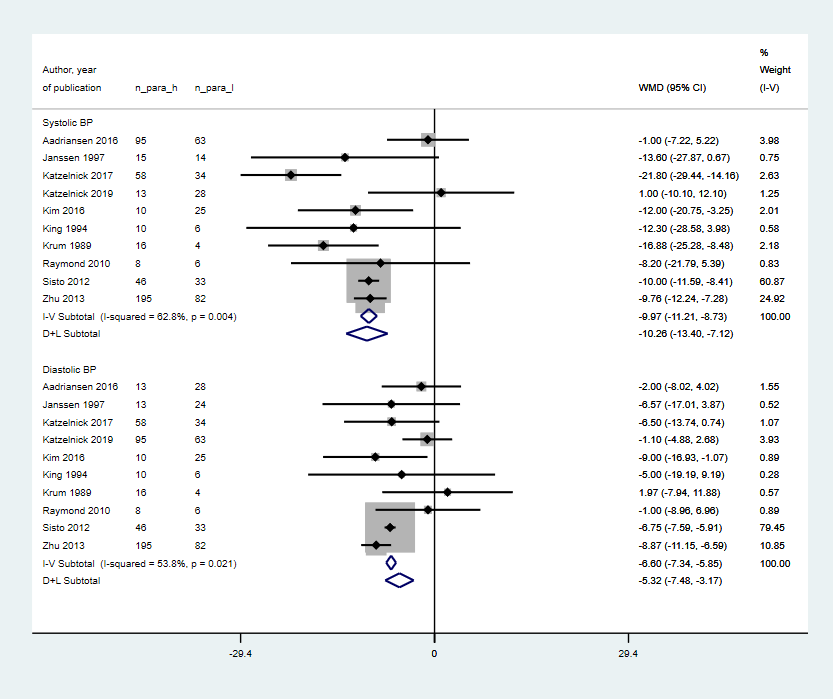  Systolic and diastolic BP (high para vs low para) | 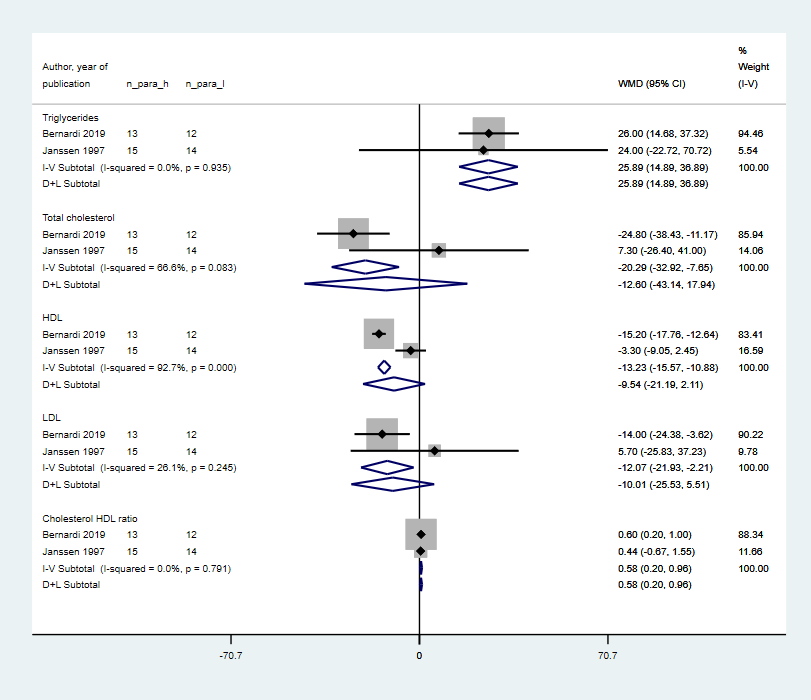  Serum lipids (high para vs low para) |

Figure A2. Leave-one out analyses

| 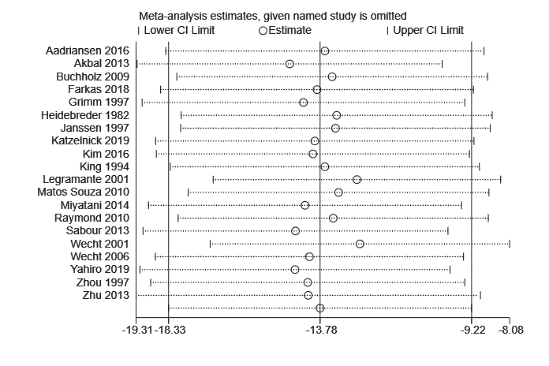  Systolic blood pressure | 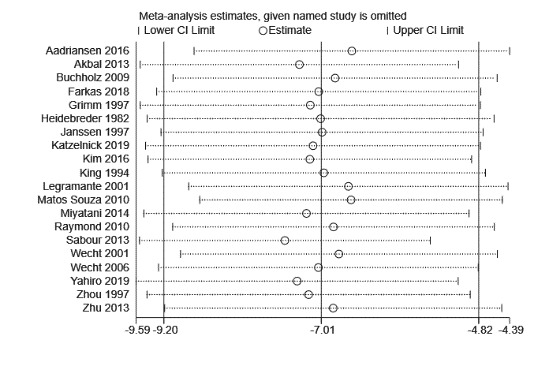  Diastolic blood pressure | 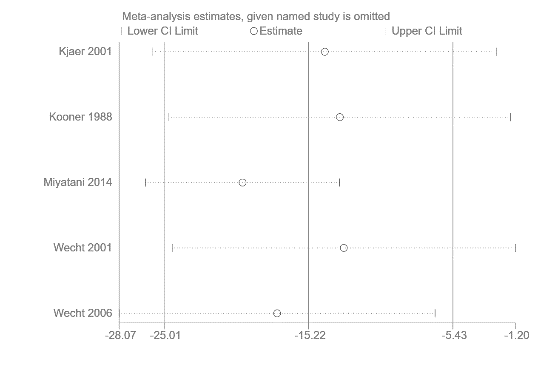  Mean arterial pressure | 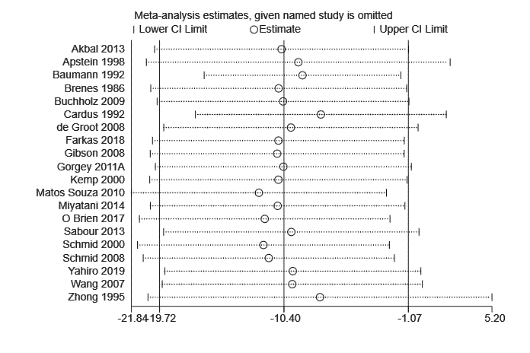Triglycerides |
| --- | --- | --- | --- |
| 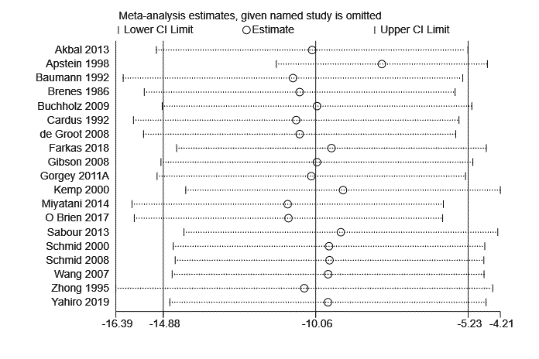  Total cholesterol | 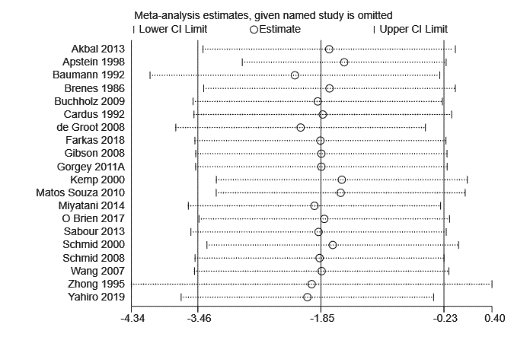  High density lipoprotein | 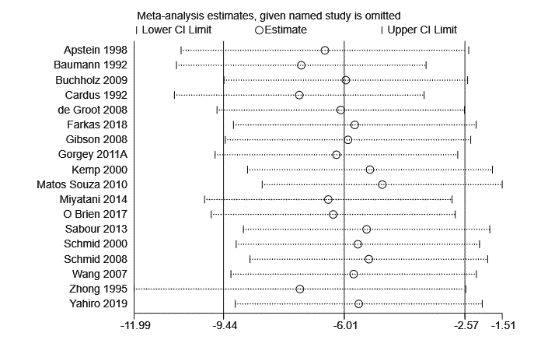Low density lipoprotein | 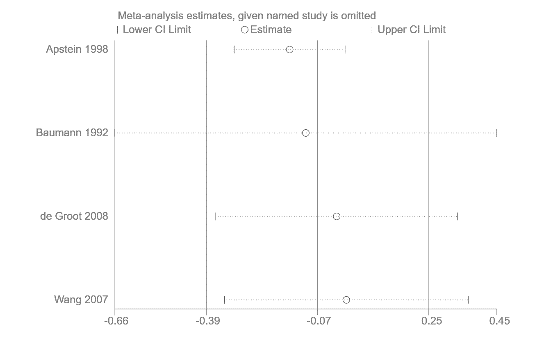  HDL LDL ratio |
| 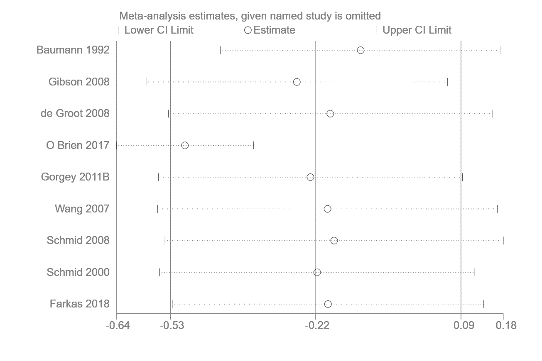  Cholesterol HDL | 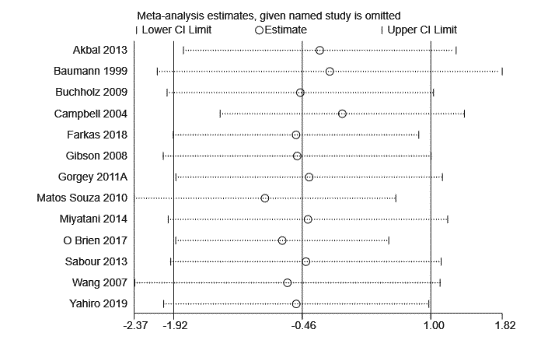  Glucose | 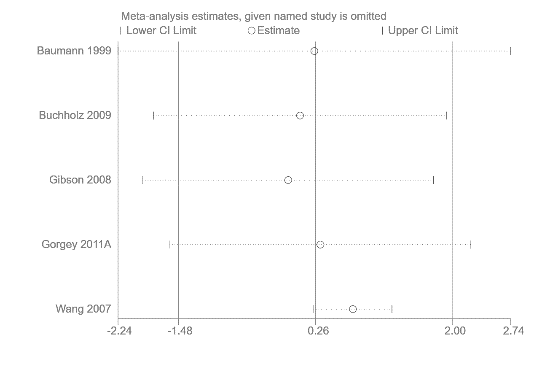  Insulin | 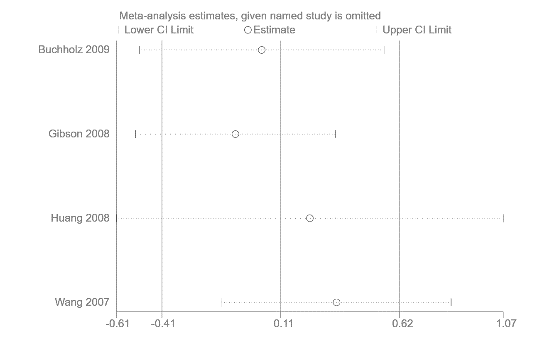  HOMA IR |
| 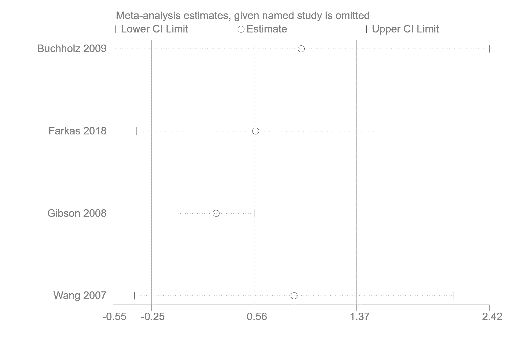  High sensitivity C reactive protein | 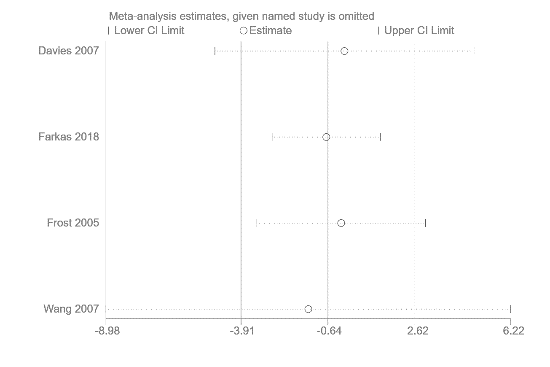  Interleukin 6 | 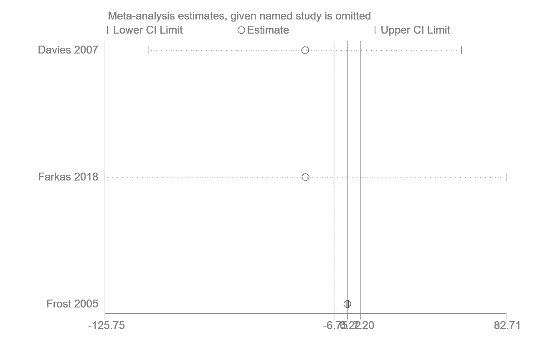  Tumor necrosis factor alpha | *Leave one out analysis recomputes the weighted mean difference upon removing one study, iteratively. This plot shows the studies removed (y-axis) and the recomputed mean difference/effect estimate (x-axis). Solid vertical lines represent the overall estimate/mean difference and the upper and lower limit of the complete analysis.  Each horizontal line represents the recomputed weighted mean difference upon removing the study indicated (circle as the effect estimate and bars as the confidence interval). |

Figure A3. Tests for publication bias (funnel plot and egger’s test)

| 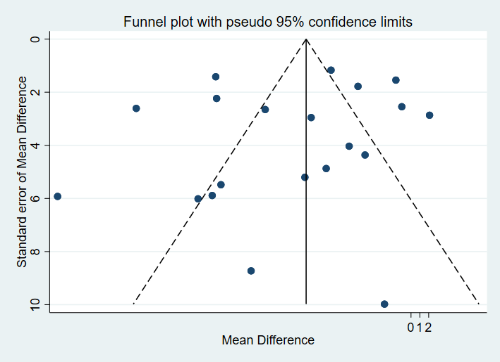  Systolic blood pressure  Egger’s test p= 0.487 | 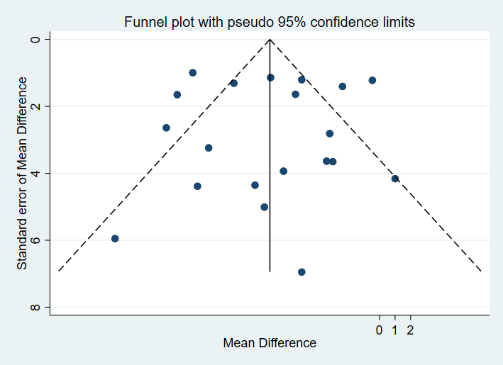  Diastolic blood pressure  Egger’s test p= 0.961 | 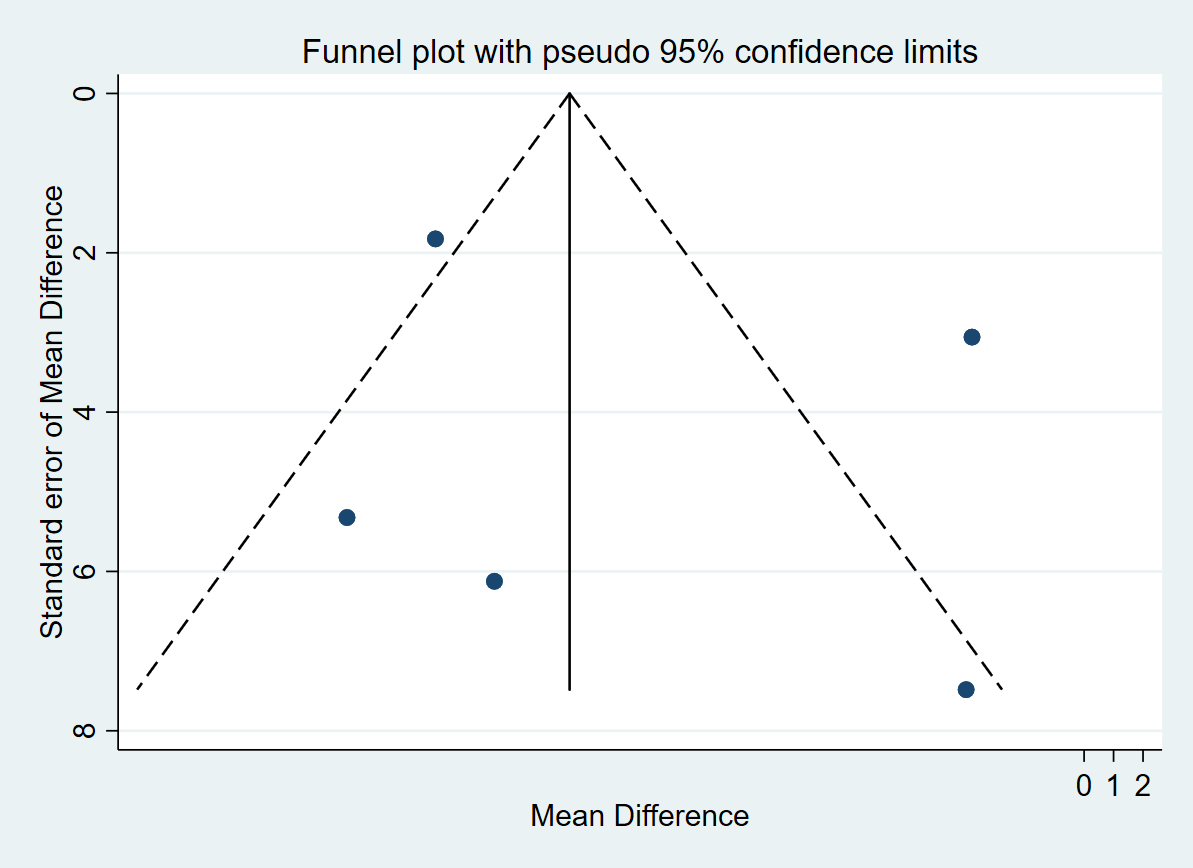 Mean arterial pressure  Egger’s test p= 0.607 | 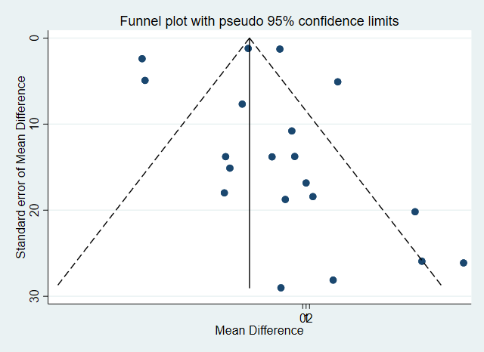  Triglycerides  Egger’s test p= 0.795 |
| --- | --- | --- | --- |
| 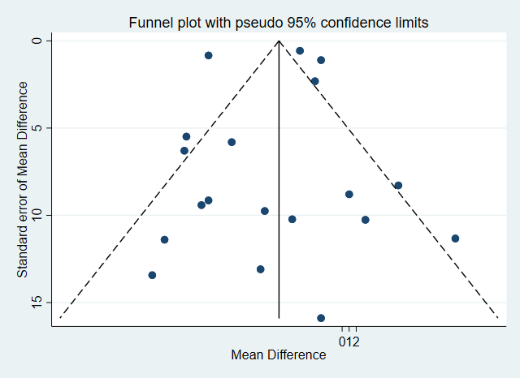  Total cholesterol  Egger’s test p= P = 0.819 | 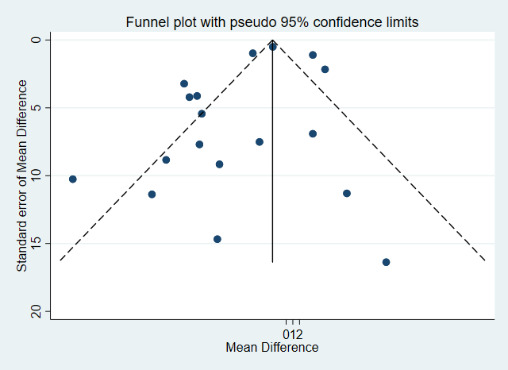  Low density lipoprotein  Egger’s test p= 0.166 | 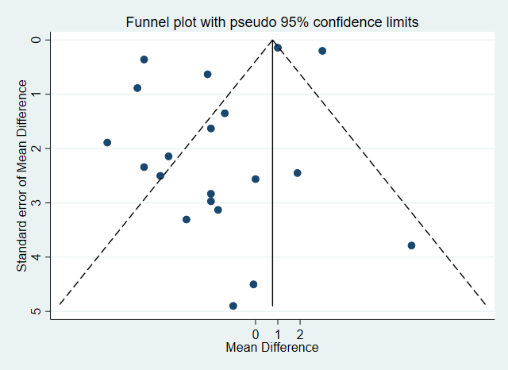  High density lipoprotein  Egger’s test p= 0.102 | 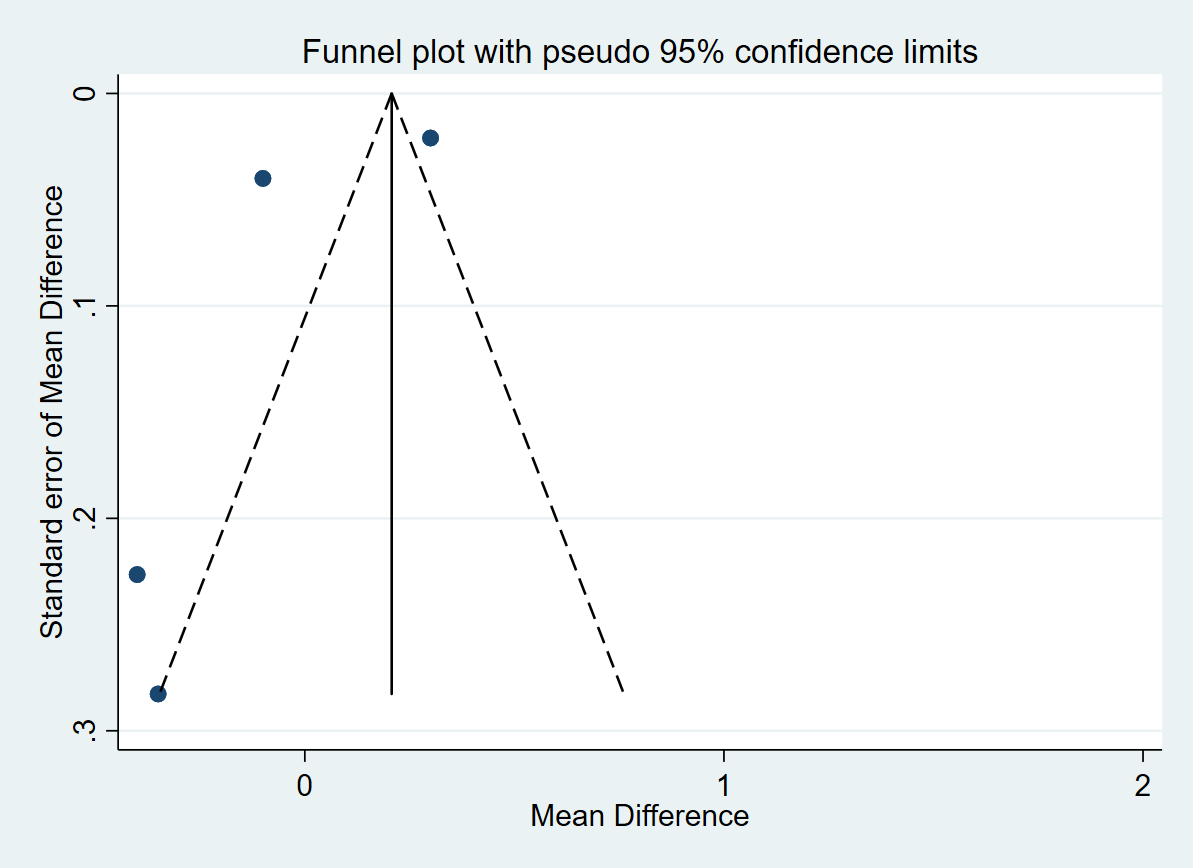 HDL LDL ratio  Egger’s test p=0.374 |
| 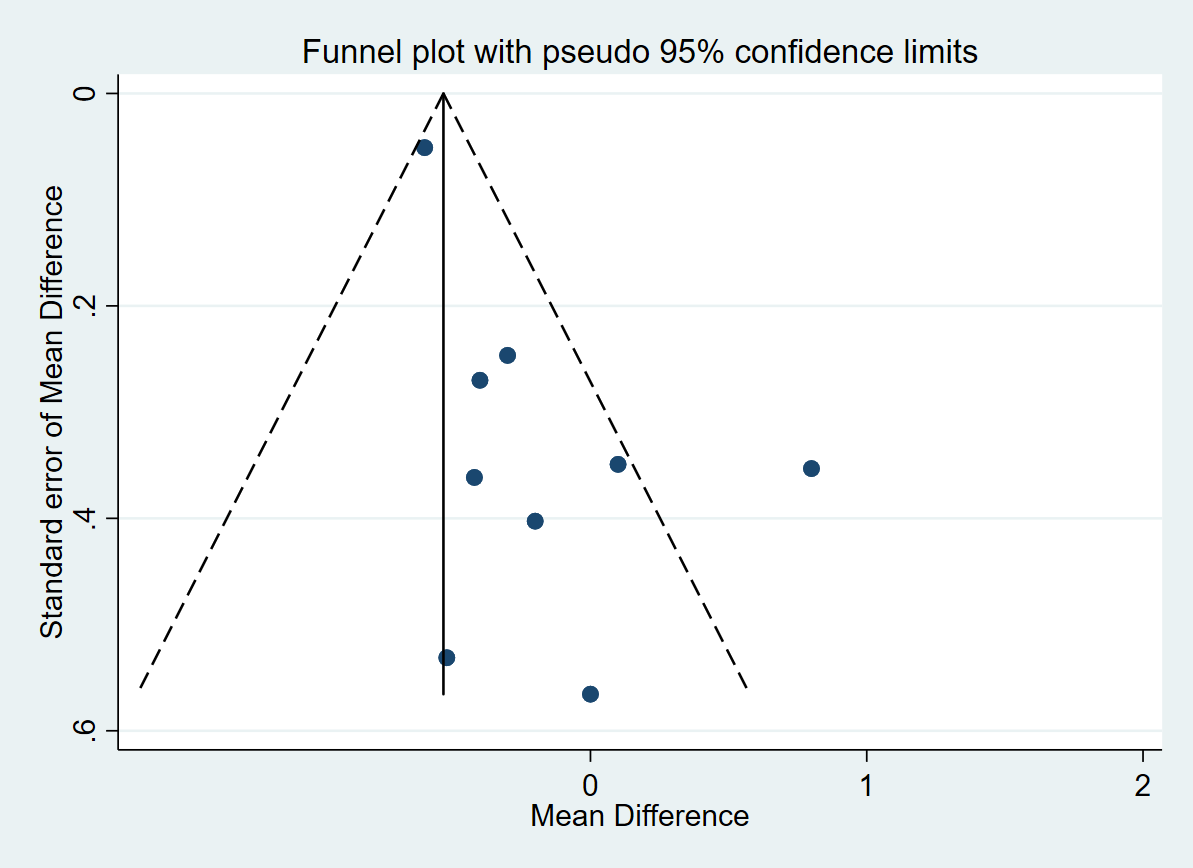 Cholesterol HDL ratio  Egger’s test p= 0.025 | 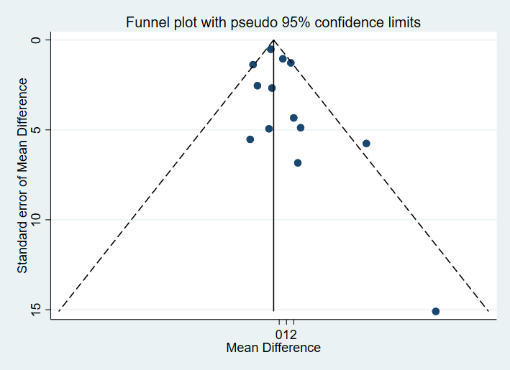  Glucose  Egger’s test p= 0.213 | 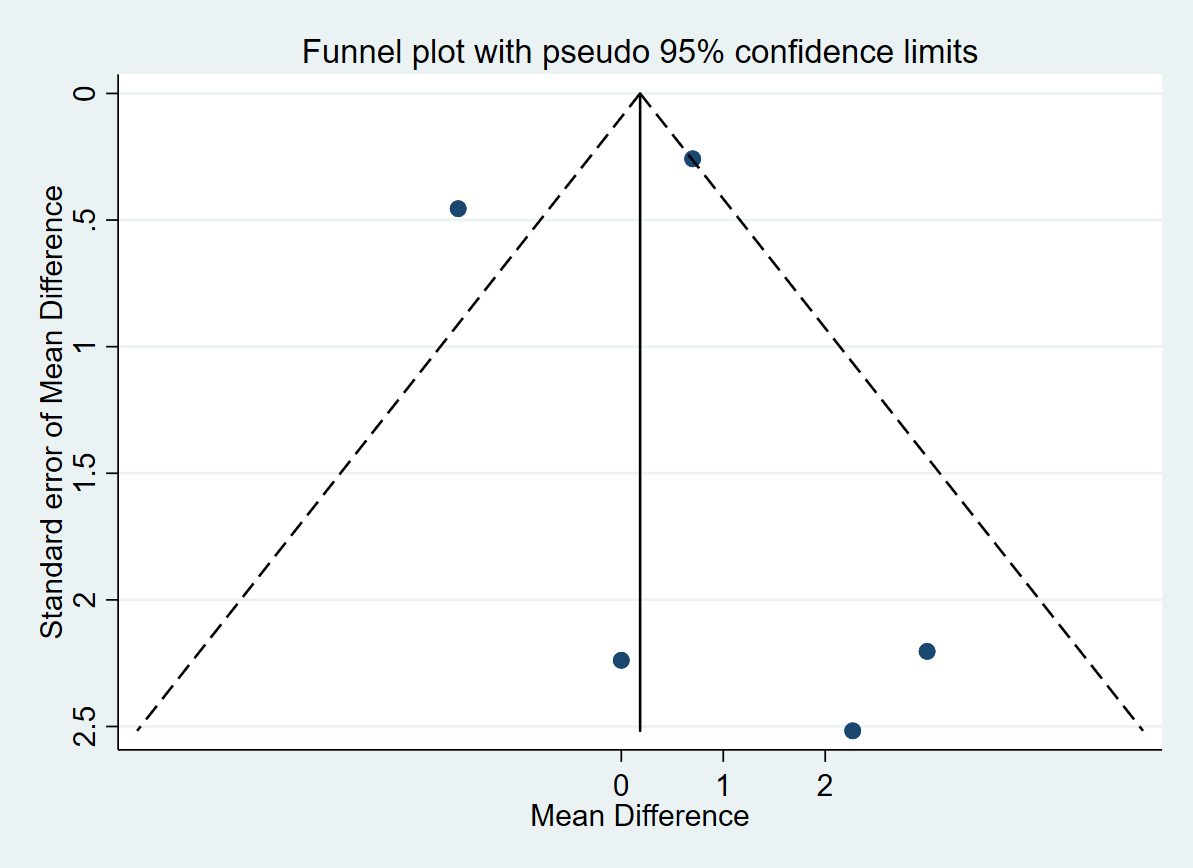 Insulin  Egger’s test p= 0.982 | 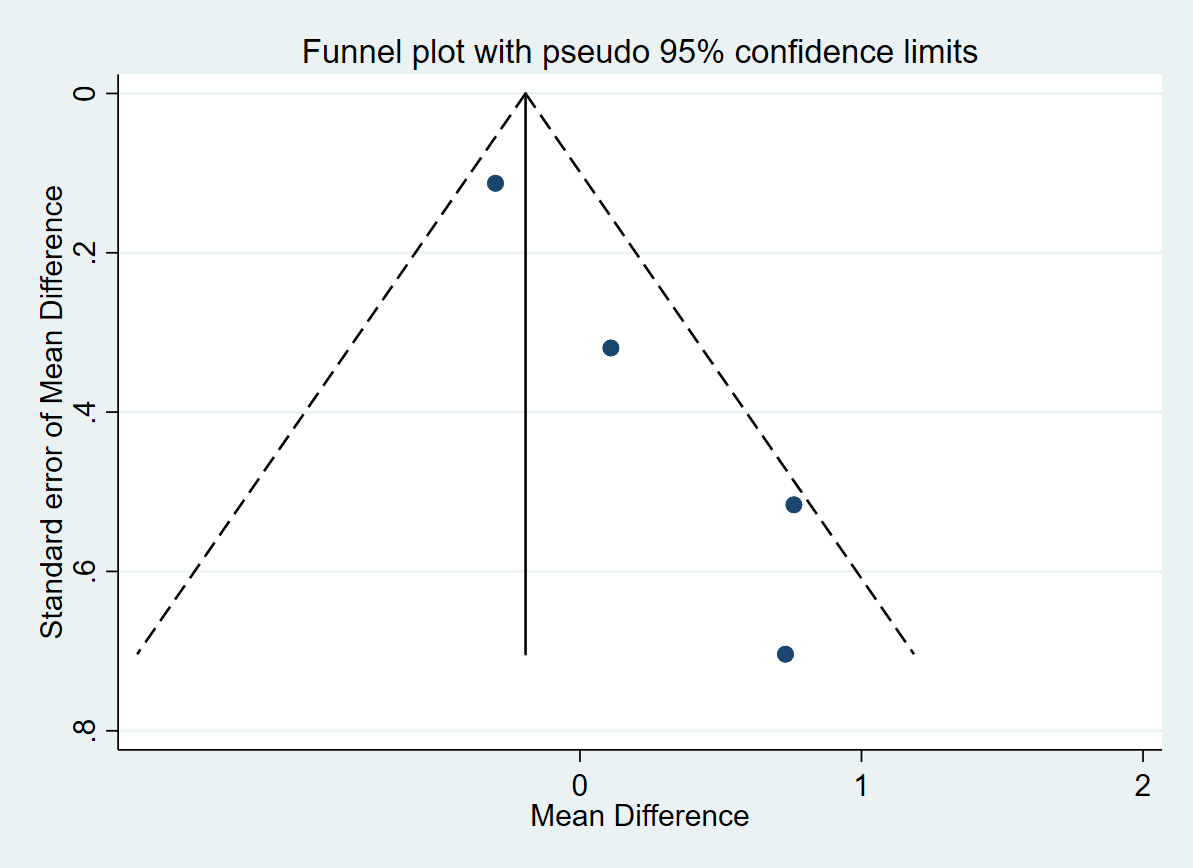 HOMA IR  Egger’s test p= 0.019 |
| 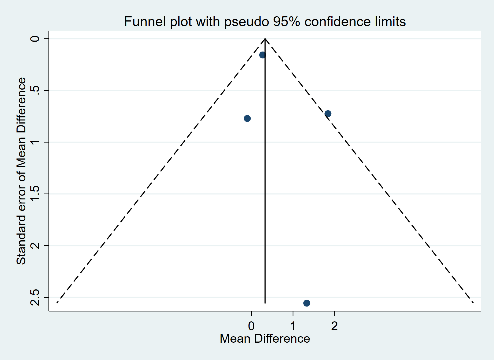  C reactive protein  Egger’s test p= 0.502 | 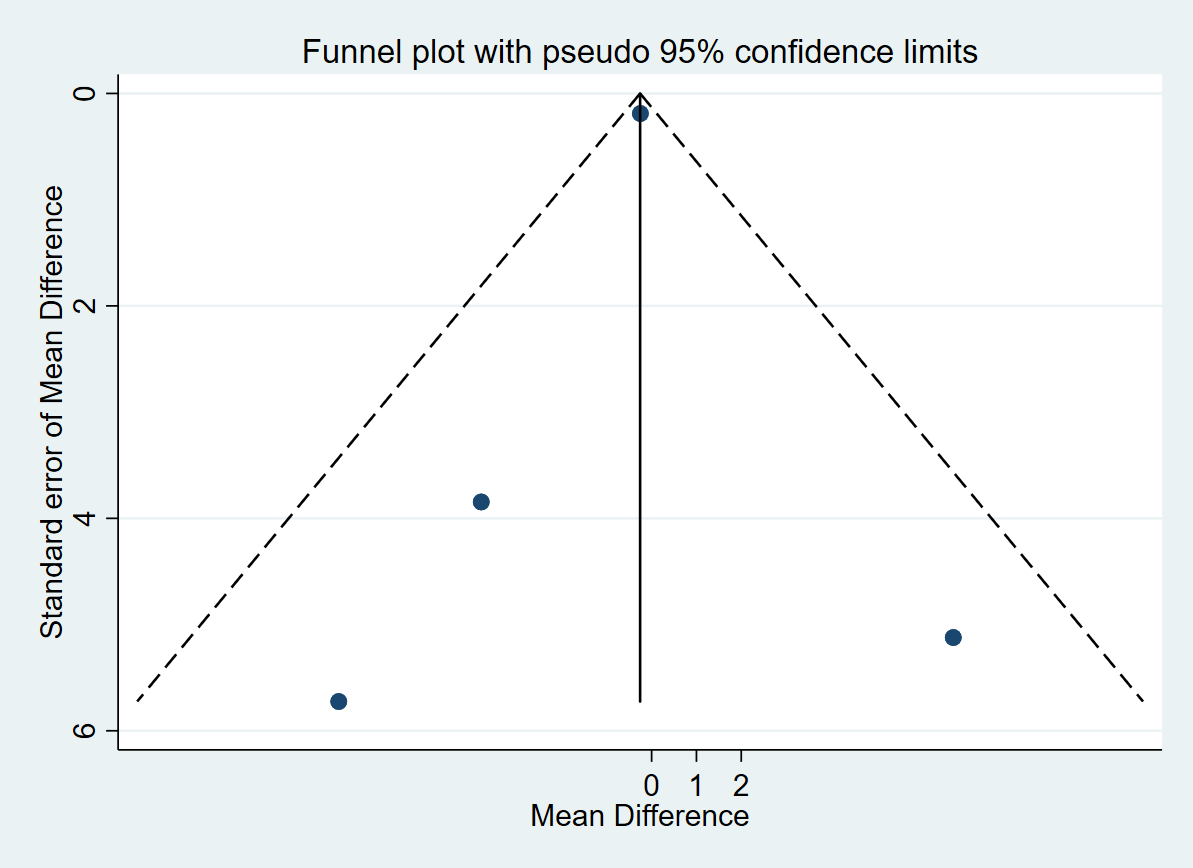 Inerleukin 6  Egger’s test p= 0.792 | 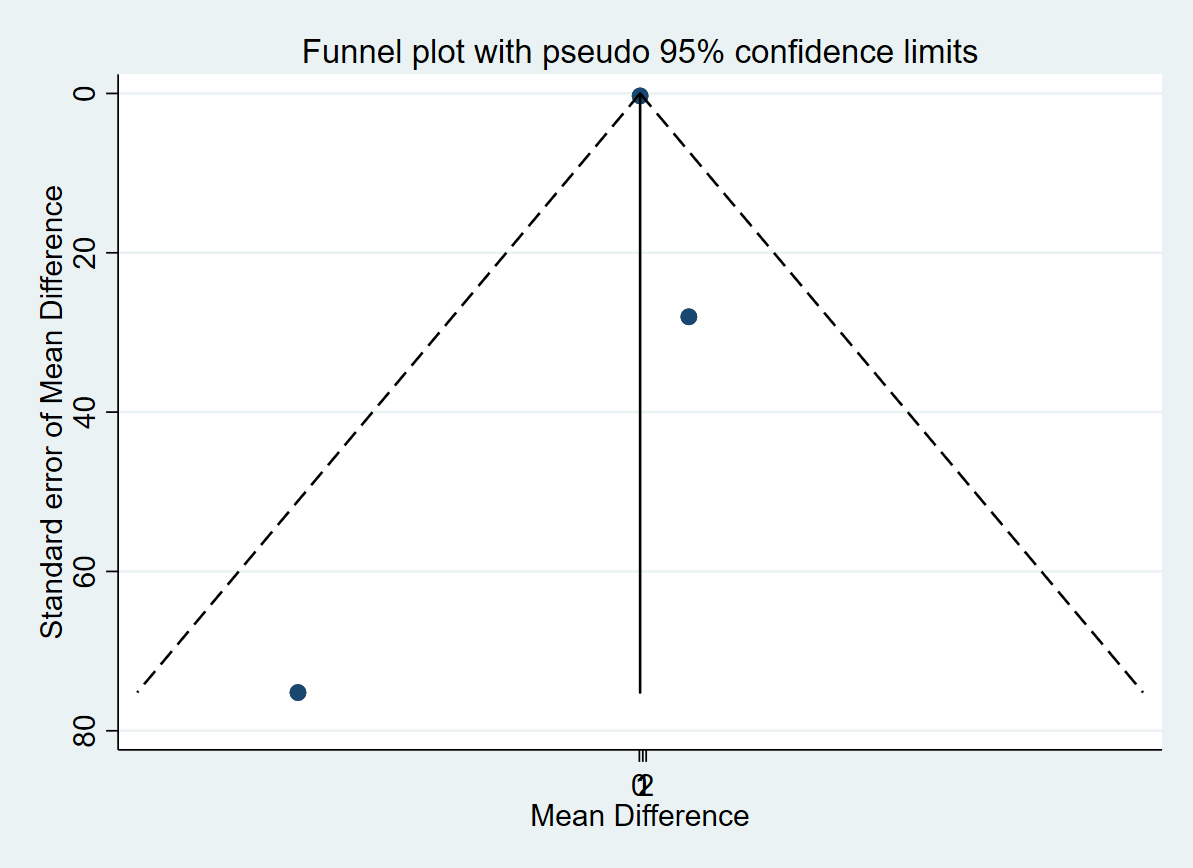 Tumor necrosis factor alpha  Egger’s test p= 0.730 | *Funnel plot visually illustrate the possibility of missing studies (publication bias) by plotting the standard error of mean difference (y axis) and the mean difference (x-axis). The dotted lines illustrate the area in which the studies (or expected studies) lies if there is no heterogeniety and biases. |

**References**

1. Adriaansen JJ, Douma-Haan Y, van Asbeck FW, van Koppenhagen CF, de Groot S, Smit CA, et al. Prevalence of hypertension and associated risk factors in people with long-term spinal cord injury living in the Netherlands. Disabil Rehabil. 2017;39(9):919-27.

2. Akbal A, Kurtaran A, Selcuk B, Akyuz M. H-FABP, cardiovascular risk factors, and functional status in asymptomatic spinal cord injury patients. Herz. 2013;38(6):629-35.

3. Apstein MD, George BC. Serum lipids during the first year following acute spinal cord injury. Metabolism. 1998;47(4):367-70.

4. Bauman WA, Adkins RH, Spungen AM, Waters RL. The effect of residual neurological deficit on oral glucose tolerance in persons with chronic spinal cord injury. Spinal Cord. 1999;37(11):765-71.

5. Bauman WA, Spungen AM, Zhong YG, Rothstein JL, Petry C, Gordon SK. Depressed serum high density lipoprotein cholesterol levels in veterans with spinal cord injury. Paraplegia. 1992;30(10):697-703.

6. Bernardi M, Fedullo AL, Di Giacinto B, Squeo MR, Aiello P, Dante D, et al. Cardiovascular Risk Factors and Haematological Indexes of Inflammation in Paralympic Athletes with Different Motor Impairments. Oxid Med Cell Longev. 2019;2019:6798140.

7. Brenes G, Dearwater S, Shapera R, LaPorte RE, Collins E. High density lipoprotein cholesterol concentrations in physically active and sedentary spinal cord injured patients. Arch Phys Med Rehabil. 1986;67(7):445-50.

8. Buchholz AC, Martin Ginis KA, Bray SR, Craven BC, Hicks AL, Hayes KC, et al. Greater daily leisure time physical activity is associated with lower chronic disease risk in adults with spinal cord injury. Appl Physiol Nutr Metab. 2009;34(4):640-7.

9. Campbell IG, Williams C, Lakomy HK. Physiological and metabolic responses of wheelchair athletes in different racing classes to prolonged exercise. J Sports Sci. 2004;22(5):449-56.

10. Cardus D, Ribas-Cardus F, McTaggart WG. Lipid profiles in spinal cord injury. Paraplegia. 1992;30(11):775-82.

11. Davies AL, Hayes KC, Dekaban GA. Clinical correlates of elevated serum concentrations of cytokines and autoantibodies in patients with spinal cord injury. Arch Phys Med Rehabil. 2007;88(11):1384-93.

12. de Groot S, Dallmeijer AJ, Post MW, Angenot EL, van der Woude LH. The longitudinal relationship between lipid profile and physical capacity in persons with a recent spinal cord injury. Spinal Cord. 2008;46(5):344-51.

13. Farkas GJ, Gorgey AS, Dolbow DR, Berg AS, Gater DR. The influence of level of spinal cord injury on adipose tissue and its relationship to inflammatory adipokines and cardiometabolic profiles. J Spinal Cord Med. 2018;41(4):407-15.

14. Frost F, Roach MJ, Kushner I, Schreiber P. Inflammatory C-reactive protein and cytokine levels in asymptomatic people with chronic spinal cord injury. Arch Phys Med Rehabil. 2005;86(2):312-7.

15. Gibson AE, Buchholz AC, Martin Ginis KA, Group S-SR. C-Reactive protein in adults with chronic spinal cord injury: increased chronic inflammation in tetraplegia vs paraplegia. Spinal Cord. 2008;46(9):616-21.

16. Gorgey AS, Gater DR. Regional and relative adiposity patterns in relation to carbohydrate and lipid metabolism in men with spinal cord injury. Appl Physiol Nutr Metab. 2011;36:107-14.

17. Gorgey AS, Gater DR. A preliminary report on the effects of the level of spinal cord injury on the association between central adiposity and metabolic profile. PM R. 2011;3(5):440-6.

18. Grimm DR, De Meersman RE, Almenoff PL, Spungen AM, Bauman WA. Sympathovagal balance of the heart in subjects with spinal cord injury. The American journal of physiology. 1997;272(2 Pt 2):H835-42.

19. Heidbreder E, Ziegler A, Heidland A, Kirsten R, Gruninger W. Circulatory changes during mental stress in tetraplegic and paraplegic man. Klinische Wochenschrift. 1982;60(15):795-801.

20. Huang CC, Liu CW, Weng MC, Chen TW, Huang MH. Association of C-reactive protein and insulin resistance in patients with chronic spinal cord injury. J Rehabil Med. 2008;40(10):819-22.

21. Janssen TW, van Oers CA, van Kamp GJ, TenVoorde BJ, van der Woude LH, Hollander AP. Coronary heart disease risk indicators, aerobic power, and physical activity in men with spinal cord injuries. Arch Phys Med Rehabil. 1997;78(7):697-705.

22. Katzelnick CG, Weir JP, Chiaravalloti ND, Wylie GR, Dyson-Hudson TA, Bauman WA, et al. Impact of Blood Pressure, Lesion Level, and Physical Activity on Aortic Augmentation Index in Persons with Spinal Cord Injury. J Neurotrauma. 2017;34(24):3407-15.

23. Katzelnick CG, Weir JP, Jones A, Galea M, Dyson-Hudson TA, Kirshblum SC, et al. Blood Pressure Instability in Persons With SCI: Evidence From a 30-Day Home Monitoring Observation. Am J Hypertens. 2019;32(10):938-44.

24. Kemp BJ, Spungen AM, Adkins RH, Krause JS, Bauman WA. The relationships among serum lipid levels, adiposity, and depressive symptomatology in persons aging with spinal cord injury. J Spinal Cord Med. 2000;23(4):216-20.

25. Kim JH, Trilk JL, Smith R, Asif I, Maddux PT, Ko YA, et al. Cardiac Structure and Function in Elite Para-cyclists with Spinal Cord Injury. Med Sci Sports Exerc. 2016;48(8):1431-7.

26. King ML, Lichtman SW, Pellicone JT, Close RJ, Lisanti P. Exertional hypotension in spinal cord injury. Chest. 1994;106(4):1166-71.

27. Kjaer M, Mohr T, Dela F, Secher N, Galbo H, Olesen H, et al. Leg uptake of calcitonin gene-related peptide during exercise in spinal cord injured humans. Clinical physiology (Oxford, England). 2001;21(1):32-8.

28. Kooner JS, Frankel HL, Mirando N, Peart WS, Mathias CJ. Haemodynamic, hormonal and urinary responses to postural change in tetraplegic and paraplegic man. Paraplegia. 1988;26(4):233-7.

29. Krum H, Howes LG, Brown DJ, Louis WJ. Blood pressure variability in tetraplegic patients with autonomic hyperreflexia. Paraplegia. 1989;27(4):284-8.

30. Legramante JM, Raimondi G, Massaro M, Iellamo F. Positive and negative feedback mechanisms in the neural regulation of cardiovascular function in healthy and spinal cord-injured humans. Circulation. 2001;103(9):1250-5.

31. Laclaustra M, Van Den Berg EL, Hurtado-Roca Y, Castellote JM. Serum lipid profile in subjects with traumatic spinal cord injury. PLoS One. 2015;10(2):e0115522.

32. Matos-Souza JR, Pithon KR, Ozahata TM, Oliveira RT, Teo FH, Blotta MH, et al. Subclinical atherosclerosis is related to injury level but not to inflammatory parameters in spinal cord injury subjects. Spinal Cord. 2010;48(10):740-4.

33. Miyatani M, Szeto M, Moore C, Oh PI, McGillivray CF, Catharine Craven B. Exploring the associations between arterial stiffness and spinal cord impairment: A cross-sectional study. J Spinal Cord Med. 2014;37(5):556-64.

34. O'Brien LC, Chen Q, Savas J, Lesnefsky EJ, Gorgey AS. Skeletal muscle mitochondrial mass is linked to lipid and metabolic profile in individuals with spinal cord injury. Eur J Appl Physiol. 2017;117(11):2137-47.

35. Raymond J, Harmer AR, Temesi J, van Kemenade C. Glucose tolerance and physical activity level in people with spinal cord injury. Spinal Cord. 2010;48(8):591-6.

36. Sabour H, Javidan AN, Ranjbarnovin N, Vafa MR, Khazaeipour Z, Ghaderi F, et al. Cardiometabolic risk factors in Iranians with spinal cord injury: analysis by injury-related variables. J Rehabil Res Dev. 2013;50(5):635-42.

37. Schmid A, Halle M, Stutzle C, Konig D, Baumstark MW, Storch MJ, et al. Lipoproteins and free plasma catecholamines in spinal cord injured men with different injury levels. Clin Physiol. 2000;20(4):304-10.

38. Schmid A, Knoebber J, Vogt S, Konig D, Deibert P, Bultermann D, et al. Lipid profiles of persons with paraplegia and tetraplegia: sex differences. J Spinal Cord Med. 2008;31(3):285-9.

39. Sisto SA, Lorenz DJ, Hutchinson K, Wenzel L, Harkema SJ, Krassioukov A. Cardiovascular status of individuals with incomplete spinal cord injury from 7 NeuroRecovery Network rehabilitation centers. Arch Phys Med Rehabil. 2012;93(9):1578-87.

40. Wang TD, Wang YH, Huang TS, Su TC, Pan SL, Chen SY. Circulating levels of markers of inflammation and endothelial activation are increased in men with chronic spinal cord injury. J Formos Med Assoc. 2007;106(11):919-28.

41. Wecht JM, De Meersman RE, Weir JP, Spungen AM, Bauman WA, Grimm DR. The effects of autonomic dysfunction and endurance training on cardiovascular control. Clinical autonomic research : official journal of the Clinical Autonomic Research Society. 2001;11(1):29-34.

42. Wong PY, Wang YC, Chu NK, Tang FT, Wong MK. Body weight, serum uric acid and lipid profile one year after spinal cord injury. Chang Gung Med J. 2001;24(9):569-75.

43. Wecht JM, Weir JP, Bauman WA. Blunted heart rate response to vagal withdrawal in persons with tetraplegia. Clinical autonomic research : official journal of the Clinical Autonomic Research Society. 2006;16(6):378-83.

44. Yahiro A, Wingo B, Kunwor S, Parton J, Ellis A. Classification of obesity, cardiometabolic risk, and metabolic syndrome in adults with spinal cord injury. J Spinal Cord Med. 2019;43(4):485-96.

45. Zhong YG, Levy E, Bauman WA. The relationships among serum uric acid, plasma insulin, and serum lipoprotein levels in subjects with spinal cord injury. Horm Metab Res. 1995;27(6):283-6.

46. Zhou GC, Yu J, Tang HH, Shi J. The determination of vasoactive substances during autonomic dysreflexia. Spinal Cord. 1997;35(6):390-3.

47. Zhu C, Galea M, Livote E, Signor D, Wecht JM. A retrospective chart review of heart rate and blood pressure abnormalities in veterans with spinal cord injury. J Spinal Cord Med. 2013;36(5):463-75.

48. Campagnolo DI, Bartlett JA, Keller SE. Influence of neurological level on immune function following spinal cord injury: a review. J Spinal Cord Med. 2000;23(2):121-8.

49. Groah SL, Nash MS, Ward EA, Libin A, Mendez AJ, Burns P, et al. Cardiometabolic risk in community-dwelling persons with chronic spinal cord injury. J Cardiopulm Rehabil Prev. 2011;31(2):73-80.

50. Kamakshi R. Assess Cardiovascular Risk in cases of Spinal Cord Injury by screening Impaired Glucose Tolerance and Dyslipidemia: Cross Sectional Study. Tamil Nadu, India: Tamil Nadu Dr.M.G.R. Medical University; 2019.

51. Kanyilmaz S, Hepguler S, Atamaz FC, Gokmen NM, Ardeniz O, Sin A. Phagocytic and oxidative burst activity of neutrophils in patients with spinal cord injury. Arch Phys Med Rehabil. 2013;94(2):369-74.

52. Kliesch WF, Cruse JM, Lewis RE, Bishop GR, Brackin B, Lampton JA. Restoration of depressed immune function in spinal cord injury patients receiving rehabilitation therapy. Paraplegia. 1996;34(2):82-90.

53. Krum H, Howes LG, Brown DJ, Ungar G, Moore P, McNeil JJ, et al. Risk factors for cardiovascular disease in chronic spinal cord injury patients. Paraplegia. 1992;30(6):381-8.

54. Lee MY, Myers J, Abella J, Froelicher VF, Perkash I, Kiratli BJ. Homocysteine and hypertension in persons with spinal cord injury. Spinal Cord. 2006;44(8):474-9.

55. Lee YH, Lee JH, Kim SH, Yi D, Oh KJ, Kim JH, et al. Hemodynamic Adaptations to Regular Exercise in People With Spinal Cord Injury. Ann. 2017;41(1):25-33.

56. Liang H, Mojtahedi MC, Chen D, Braunschweig CL. Elevated C-reactive protein associated with decreased high-density lipoprotein cholesterol in men with spinal cord injury. Arch Phys Med Rehabil. 2008;89(1):36-41.

57. Maruyama Y, Mizuguchi M, Yaginuma T, Kusaka M, Yoshida H, Yokoyama K, et al. Serum leptin, abdominal obesity and the metabolic syndrome in individuals with chronic spinal cord injury. Spinal Cord. 2008;46(7):494-9.

58. Ozkul I, Unalan H, Erhan B, Damci T, Uludag M, Can G. Glucose Intolerance, Insulin Resistance, and C Peptide Levels in Patients With Spinal Cord Injury. Neurosurgery Quarterly. 2007;17(3):170-4.

59. Rankin KC, O'Brien LC, Segal L, Khan MR, Gorgey AS. Liver Adiposity and Metabolic Profile in Individuals with Chronic Spinal Cord Injury. Biomed Res Int. 2017;2017:1364818.

60. Storch MJ, Konig D, Bultermann D, Blum A, Vogt S, Baumstark M, et al. Lipid profile in spinal cord-injured women with different injury levels. Prev Med. 2005;40(3):321-5.

61. Vidal J, Javierre C, Curia FJ, Garrido E, Lizarraga MA, Segura R. Long-term evolution of blood lipid profiles and glycemic levels in patients after spinal cord injury. Spinal Cord. 2003;41(3):178-81.

62. Wang YH, Chen SY, Wang TD, Hwang BS, Huang TS, Su TC. The relationships among serum glucose, albumin concentrations and carotid atherosclerosis in men with spinal cord injury. Atherosclerosis. 2009;206(2):528-34.

63. Vaziri ND, Gordon S, Nikakhtar B. Lipid abnormalities in chronic renal failure associated with spinal cord injury. Paraplegia. 1982;20(3):183-9.

64. Yamamotova A, Sramkova T, Rokyta R. Intensity of pain and biochemical changes in blood plasma in spinal cord trauma. Spinal Cord. 2010;48(1):21-6.

65. Yoo KY, Jeong SW, Kim SJ, Ha IH, Lee J. Cardiovascular responses to endotracheal intubation in patients with acute and chronic spinal cord injuries. Anesthesia and analgesia. 2003;97(4):1162-7, table of contents.
